# 1100 synthetic benchmark problems for dynamic modeling of cellular processes

**DOI:** 10.1093/bioadv/vbag307

**Published:** 2026-10-10

**Authors:** Niklas Neubrand, Timo Rachel, Tim Litwin, Jens Timmer, Clemens Kreutz, Moritz Hess

**Affiliations:** Institute of Medical Biometry and Statistics, Faculty of Medicine and Medical Center, University of Freiburg, Freiburg 79104, Germany; Institute of Medical Biometry and Statistics, Faculty of Medicine and Medical Center, University of Freiburg, Freiburg 79104, Germany; Institute of Medical Biometry and Statistics, Faculty of Medicine and Medical Center, University of Freiburg, Freiburg 79104, Germany; Center for Data Analysis, Modeling and AI (FDMAI), University of Freiburg, Freiburg 79104, Germany; Center for Data Analysis, Modeling and AI (FDMAI), University of Freiburg, Freiburg 79104, Germany; Institute of Physics, University of Freiburg, Freiburg 79104, Germany; Centre for Integrative Biological Signalling Studies (CIBSS), University of Freiburg, Freiburg 79104, Germany; Institute of Medical Biometry and Statistics, Faculty of Medicine and Medical Center, University of Freiburg, Freiburg 79104, Germany; Institute of Medical Biometry and Statistics, Faculty of Medicine and Medical Center, University of Freiburg, Freiburg 79104, Germany

## Abstract

**Motivation:**

Systems biology strives to unravel the complex dynamics of cellular processes using ordinary differential equations. However, the sparsity of measured data and the strong nonlinearity of common ordinary differential equations introduce severe numerical problems in modeling tasks. This motivated the development of computational algorithms that must be systematically evaluated to ensure optimal method choices. The number of well-curated models for such benchmarking efforts is insufficient, as building and calibrating biologically reasonable models from experiments requires years of work.

**Results:**

We present a large-scale collection of 1100 synthetic modeling problems, generated based on the systems of ordinary differential equations and experimental designs of 22 published modeling problems. This is achieved by extending a recent method for simulation of time-course data for randomly generated observation functions to also include realistic measurement patterns across multiple experimental conditions. By analyzing data and model characteristics, optimization performance, and parameter identifiability, we show that the synthetic problems provide both a realistic and diverse extension of the existing problem space. Hence, the synthetic collection provides a valuable resource for benchmarking in dynamic modeling.

**Availability and implementation:**

Benchmark problems and algorithm are publicly available at https://github.com/niklasneubrand/1100SyntheticBenchmarksODE and https://doi.org/10.5281/zenodo.23211070.

## 1 Introduction

Dynamic modeling based on ordinary differential equations (ODEs) is a fundamental technique in systems biology (Kitano, 2002, Qiao *et al.* 2025). Such mechanistic models describe biochemical reaction networks underlying cellular processes such as signaling pathways. They include unknown parameters (e.g. reaction rate constants, Hill coefficients, or initial values) that must be inferred from experimental data (Raue *et al.* 2013, Villaverde *et al.* 2022). To dissect the roles of individual system components, models are analyzed across multiple experimental conditions—for instance, with different stimuli or genetic perturbations. In each condition, distinct observables (i.e. measurable quantities such as protein concentrations or phosphorylation states) can be recorded, depending on experimental feasibility. This variety gives rise to complex data structures and intricate mappings between the observations and the underlying ODE equations (Raue et al. 2013, Villaverde *et al.* 2022).

To manage this complexity of real-world modeling problems, several dedicated computational toolboxes have been developed. Examples include Data2Dynamics (Raue *et al.* 2015), dMod (Kaschek *et al.* 2019), pyPESTO (Schälte *et al.* 2023), and PEtab.jl (Persson *et al.* 2025). These environments typically rely on standardized, human-readable specifications such as the Data2Dynamics or PEtab formats (Schmiester *et al.* 2021). Together, they provide integrated frameworks for model simulation, parameter calibration, and uncertainty quantification, supporting both frequentist (Kreutz *et al.* 2012, 2013) and Bayesian approaches (Schälte *et al.* 2023). These frameworks also enable higher-level modeling tasks such as model reduction (Maiwald *et al.* 2016) or automated model selection (Pathirana *et al.* 2026).

However, despite this extensive tool support, modeling projects are often complicated by numerical challenges. Solving stiff and high-dimensional ODE systems can be computationally demanding or even unfeasible (Kreutz 2019, Villaverde *et al.* 2022). Moreover, the nonlinearity of ODE solutions gives rise to objective functions with nonconvex regions and multiple local optima, while sparse or noisy experimental data can induce flat directions in the parameter space, termed nonidentifiability (Raue et al. 2009, Wieland *et al.* 2021, Transtrum et al. 2015). As a result, parameter estimation frequently suffers from convergence issues; identifying the global optimum reliably remains challenging (Kreutz 2019, Raue *et al.* 2013).

To address these issues, it is essential to select methods and configurations that are well suited to the specific modeling problem. In practice, however, these choices are often based on experience or trial-and-error. To derive more general recommendations, systematic benchmarking across a wide range of modeling scenarios is required. While several benchmarking studies have examined particular aspects such as parameter estimation (Hass *et al.* 2019, Kreutz 2019, Villaverde *et al.* 2019) or sensitivity computation (Lakrisenko *et al.* 2024), these typically rely on small sets of ODE models or simplified synthetic problems and are therefore limited in scope. To improve and standardize such efforts, a curated collection of 20 peer-reviewed models calibrated on experimental data has been published as a general benchmark resource (Hass *et al.* 2019). For comprehensive benchmarking, such problems must include not only the ODE models themselves but also curated experimental datasets, observable definitions, and complete mappings between measurements and model variables. However, because both experimental research and mathematical model development are labor-intensive processes, the number of such fully specified modeling problems remains limited.

In this work, we apply synthetic data generation to scale up the existing benchmark collection. We build on a recent method for generating time-course (TC) data sets for randomly generated observables of a given ODE system (Egert and Kreutz 2023). To better mimic the complexities of real-world modeling problems, we extend this algorithm to work with multiple experimental conditions and develop a method to generate realistic observable patterns across experimental conditions. Central to our approach is the use of a *simulation template*, which consists of the ODE system, experimental conditions, and observation functions of an existing calibrated model. This template provides the structural and biological context from which diverse synthetic modeling problems are generated.

Using 22 published modeling problems from systems biology—including intracellular reaction networks such as JAK-STAT signaling (Swameye *et al.* 2003, Bachmann *et al.* 2011, Merkle *et al.* 2016, Sobotta *et al.* 2017) or population-level cell dynamics (Crauste *et al.* 2017)—we generate a collection of 1100 synthetic modeling problems. We observe both realism and diversity in key problem characteristics. The synthetic problems reproduce the statistical distributions of the template collection while exhibiting substantial variability within each template subgroup. Furthermore, parameter optimization and identifiability analyses show that the synthetic collection covers a broad spectrum of numerical behavior, including both well-behaved problems comparable to the templates and challenging cases with high optimization failure rates or reduced parameter identifiability. With these properties, the synthetic collection provides a valuable resource for method development and systematic benchmarking of computational tools for dynamic modeling in systems biology.

## 2 Methods

### 2.1 Dynamic modeling with ODEs

Throughout this work, we consider biological systems, e.g. metabolic networks or signaling pathways, that can be expressed by a system of ODEs


(1) $$\frac{d}{dt}\vec{x}(t)=f\left(\vec{x}(t),\vec{u}(t);{\vec{\theta }}_{\text{dyn}}\right).$$

In the systems biology context, the *dynamic variables* $\vec{x}(t)$ typically represent concentrations of molecular species; $\vec{u}(t)$ are input functions and ${\vec{\theta }}_{\text{dyn}}$ are the dynamic parameters (e.g. reaction rate constants or Hill coefficients). An approximate solution $\vec{x}(t)$ can be obtained by numerical integration of the ODEs. Initial values $\vec{x}(t_{0})$ are either known, derived from steady-state assumptions, or estimated from the data.

To simulate different biological scenarios, e.g. genetic variations or external stimuli, parameters, initial values, or input functions of the original ODEs can be replaced or modified. Each modification defines a distinct *simulation condition* and requires an independent computation of the ODE solution.

Furthermore, the biological entities described by the ODE system often cannot be measured directly. To account for this, we introduce *observation functions g*, which map internal model states to measurable quantities. Observation functions may combine dynamic variables by sums or ratios, include transformations (e.g. logarithmic), and often introduce additional *observation parameters* ${\vec{\theta }}_{\text{obs}}$ such as scaling factors, offsets, or detection limits.

Using these observation functions, we can model experimental observations $y_{i}$ (with counting index i=1,…,N) by the statistical model


(2) $$y_{i}=g_{i}\left(\vec{x}(t_{i});{\vec{\theta }}_{\text{obs}}\right)+\epsilon_{i},$$

where $t_{i}$ denotes the measurement time, $g_{i}$ the corresponding observation function, and $\epsilon_{i}∼N\left(0,\sigma_{i}^{2}\right)$ the measurement noise. For simplicity, we assume independent, normally distributed residuals. Log-normally distributed residuals can be described by logarithmic transformations of both $y_{i}$ and $g_{i}$.

The measurement uncertainty $\sigma_{i}$ is allowed to vary across data points and is often specified by an *error model* with parameters ${\vec{\theta }}_{\text{err}}$, which typically combine absolute and relative error components. Importantly, the observable and error parameters are often condition specific to account for different experimental setups, measurement techniques, and batch effects.

Together, these modeling assumptions define the likelihood function $L\left(\vec{\theta };\vec{y}\right)$, which allows joint estimation of all unknown parameters $\vec{\theta }$ by the maximum-likelihood approach


(3) $${\hat{\vec{\theta }}}_{\text{ML}}={\text{argmax}}_{\vec{\theta }}\left(L\left(\vec{\theta };\vec{y}\right)\right).$$

### 2.2 Formalizing typical data and observable structures

In published modeling problems, observation functions and simulation conditions are carefully chosen to address specific biological questions. To describe different experimental designs, we distinguish between two types of *experimental conditions*:

- A TC experiment probes the dynamic behavior of the system under a single simulation condition, with measurements taken at multiple time points.
- A *dose–response* (DR)experiment evaluates the system’s response to varying input levels. It consists of multiple simulation conditions (e.g. different doses) but collects measurements at a single time point.

Due to technical reasons, measurements of the same underlying system components may be described by multiple different observation functions. Common examples are condition-specific parameters or transformations. To have a common description for these related measurements, we use the term *observable* for the set of all observation functions sharing a unique subset of dynamic variables. For example, the functions $g_{1}(x)=x_{1}$ and $g_{2}(x)=a\cdot x_{1}$ belong to the same observable, while $g_{3}(x)=x_{1}+x_{2}$ belongs to a different one, as it includes an additional dynamic variable.

In published modeling problems, we often observe structured patterns in how observables are distributed across experiments. We formalize this structure with the boolean *experiment–observable matrix* (EOM), in which rows represent experiments and columns represent observables. The entry ${\text{EOM}}_{jk}$ indicates whether a given observable *k* is measured in a particular experiment *j*. Examples for both an experimental and a synthetic modeling problem are shown in Fig. 1a and d, respectively.

**Figure 1:**
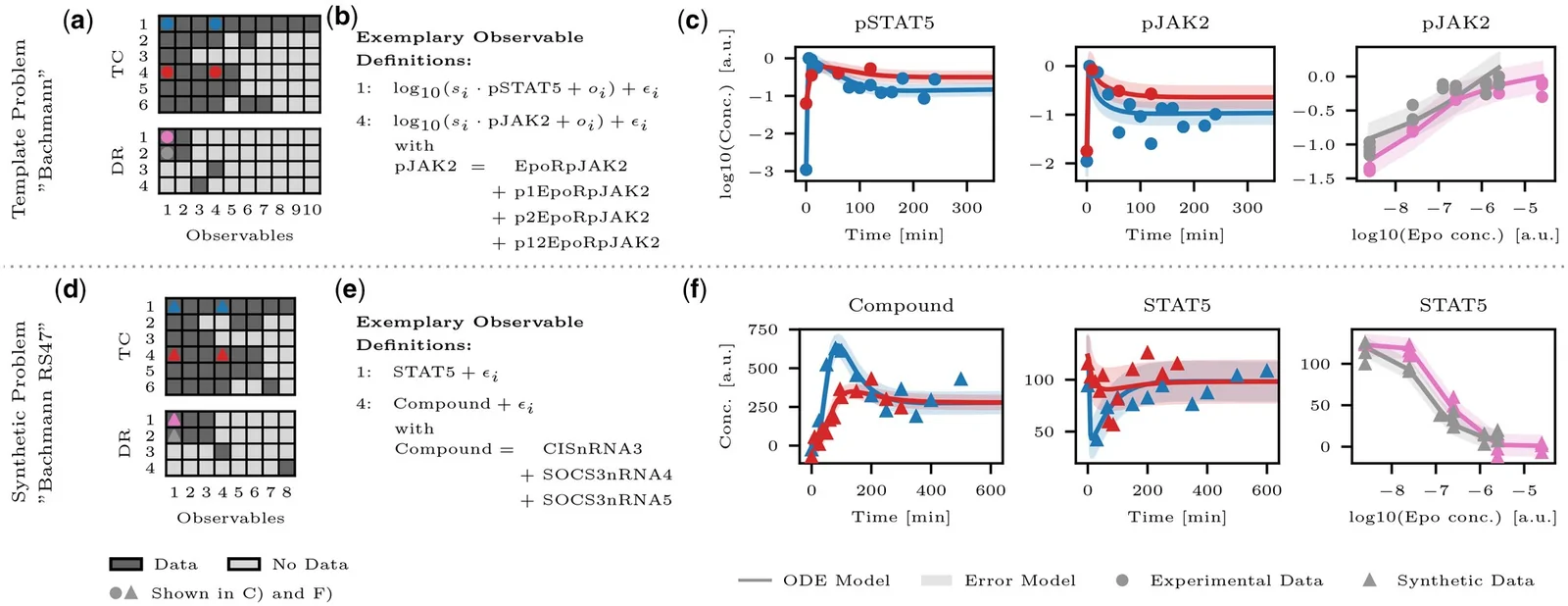
Comparison between a template problem (a–c) and a synthetic counterpart (d–f). Panels (a) and (d) show the EOM. The matrices are split into TC and DR experiments. As shown by two examples each, the observable expressions for template (b) and synthetic (e) problems differ structurally. $s_{i}$, $o_{i}$, and $\epsilon_{i}$ represent experiment-dependent scaling factors, offsets, and residual errors, respectively, while all other symbols denote dynamic ODE variables $x_{i}(t)$ (see (1)). Panels c and f show experimental (c) and synthetic (f) data for a selection of TC and DR experiments (as indicated in the EOMs) together with fitted (c) and ground-truth (f) ODE and error models. Both problems exhibit similar EOM structures, observable definitions, and data characteristics.

### 2.3 Generating realistic synthetic benchmark problems

We developed a simulation pipeline that generates realistic synthetic modeling problems based on existing modeling problems, referred to as *template problems*. Each synthetic problem is constructed in several steps:

1. Selecting simulation templates: manually choose a real-world modeling problem with known experimental structure.
2. Perturbing dynamic parameters: perturb the dynamic parameters to generate novel system behavior.
3. Generating realistic observable structures: sample biologically plausible observables from the system states and assign them realistically to experiments.
4. Generating realistic synthetic data: generate measurement time grids, simulate model output, and add biologically calibrated noise models.

These steps introduce controlled variability while retaining the biological context provided by the template models, thereby supporting the generation of realistic and diverse benchmark problems.

### 2.4 Selecting simulation templates

In our simulation study, we use a set of 22 real-world modeling problems from systems biology as simulation templates. The models describe intracellular reaction networks, e. g. the JAK-STAT signaling pathway (Swameye *et al.* 2003, Bachmann *et al.* 2011, Merkle *et al.* 2016, Sobotta *et al.* 2017), or cell population dynamics (Crauste *et al.* 2017). All models are publicly available as examples in the Data2Dynamics toolbox. Nineteen problems have already been part of the experimental benchmark collection (Hass *et al.* 2019), and three additional suitable problems were included (Borghans *et al.* 1997, Elowitz and Leibler 2000, Sneyd and Dufour 2002). In this article, we will refer to the templates by the last name of the first author. An overview table with problem characteristics and all references is given in the Supplementary material.

### 2.5 Perturbing dynamic parameters

To generate diverse system behaviors, we perturb the dynamic parameters of the simulation template. Specifically, each calibrated parameter $\theta_{i}\in {\vec{\theta }}_{\text{dyn}}$ is multiplied by a random factor $2^{\eta }$, with η∼U(−1,1). This transformation is symmetrical on the logarithmic scale and introduces realistic variability in the model dynamics.

### 2.6 Generating realistic observable structures

To generate realistic observable structures for our synthetic benchmark problems, we build on the approach of Egert and Kreutz (2023). Their method generates realistic synthetic observation functions using a sequence of random decisions, calibrated to match the observable characteristics of 19 real-world modeling problems. The resulting observable expressions are either functions of a single dynamic variable $x_{i}$ or sums over multiple variables $\sum_{i}x_{i}$—termed *compound measurements*. Additionally, the expressions may include scaling, offset parameters, or ${\;\text{log}\;}_{10}$ transformations. The number of observables is also randomly selected based on the ODE system dimension, with a typical ratio between the numbers of observable and dynamical variables of about 50% (Egert and Kreutz 2023).

We extend this approach with realistic assignments of observation functions across multiple experimental conditions. Specifically, we generate a synthetic EOM by sampling for each synthetic observable a column from the template’s EOM (uniformly with replacement). This approach transfers the template’s measurement patterns to the synthetic problem.

To avoid generating noninformative synthetic data, observation functions are only sampled from dynamic variables with time variation in at least one experiment. After sampling the synthetic EOM, we also replace the assignment of time-constant observables within each experiment to increase the time variation in the experimental design.

We emphasize that a single observable may be realized by different observation functions in different experiments. Specifically, we define individual scale, offset, and error parameters for each experiment, even when the underlying observable is shared.

### 2.7 Generating realistic synthetic data

To generate data for TC experiments, we first determine the sampling time points. For this, we use the method of Egert and Kreutz (2023), which applies the Retarded Transient Function (RTF) approach (Kreutz 2020) to analyze the dynamics of each observable. The RTF is a parametric function tailored to approximate typical ODE dynamics by combining transient and sustained dynamics. A pre-calibrated linear model then maps the RTF parameters to a realistic time grid, including the number of points, time range, and spacing.

For DR experiments, we retain the original dose levels from the template problem but introduce variability by randomizing the sampling time. Specifically, the original measurement time is scaled by a random factor $2^{\eta }$, with η∼U(−1,1).

Synthetic observations are generated by evaluating the observation functions at the chosen time points and adding Gaussian measurement noise:


(4) $$y_{i}=g_{i}(\vec{x}(t_{i});\theta )+\epsilon_{i},\;\epsilon_{i}∼N(0,\sigma_{i}^{2}({\vec{x}}_{i})).$$

If the observable is defined on a logarithmic scale, this results in log-normal noise in the data space. The noise level $\sigma_{i}({\vec{x}}_{i})$ is randomly drawn according to the model in Egert and Kreutz (2023), which was calibrated on error characteristics of 19 real-world modeling problems.

Note that scaling and offset parameters only have a minor influence on the generated data, because their ground-truth values are set to neutral values (scaling factor = 1, offset ≈ 1% of the mean value). Therefore, these parameters are mostly relevant for parameter estimation tasks.

### 2.8 Evaluation criteria

In the following, we describe the criteria and computational procedures used to evaluate the realism, complexity, and information content of the synthetic benchmark problems. We begin with basic data and model characteristics, then outline two central analysis tasks commonly encountered in dynamical modeling: multistart parameter estimation and local identifiability analysis. Finally, we perform a principal component analysis (PCA) to combine multiple metrics and to assess the structure of the full benchmark collection.

### 2.9 Data and model characteristics

To characterize each benchmark problem, we define a set of key characteristics describing both the data and the underlying statistical model. As high-level indicators of problem size and complexity, we report the number of observables, estimated parameters, and data points.

We quantify the overall noise level of each modeling problem by the median relative error. The relative error for each data point is defined as


(5) $$r_{i}=\{\begin{array}{cc} \left|\sigma_{i}\right| & \text{if}\;\text{the}\;\text{observable}\;\text{is}\;\text{on}\;a\;\;\text{log-scale},\\ \left|\sigma_{i}/y_{i}\right| & \text{if}\;\text{the}\;\text{observable}\;\text{is}\;\text{on}\;a\;\text{linear}\;\text{scale}. \end{array}$$

For synthetic problems, $y_{i}$ and $\sigma_{i}$ refer to the generated data values and ground-truth error parameters. For template problems, $y_{i}$ are experimental measurements, and $\sigma_{i}$ are either measured uncertainties or fitted error models.

In addition, we report the median measurement time to reflect both the time-scales of the system and the data sampling.

### 2.10 Multistart parameter estimation

To explore the likelihood landscape and investigate the performance of parameter estimation, we conduct a global optimization analysis using multistart local optimization. For each benchmark problem, we uniformly sample 100 random initial parameter vectors within the bound constraints. For the template, the parameter scale (linear or logarithmic) and bounds are predetermined. For synthetic problems, we use the template bounds and scales for dynamic parameters. For the novel generated error, scale, and offset parameters, we choose a logarithmic scale with bounds extending two orders of magnitude in both directions.

For each initial parameter guess, we independently perform local optimization of the likelihood function using the trust-region reflective algorithm (Coleman and Li 1996). The ODE solver uses absolute and relative tolerances of $10^{-8}$ to ensure numerical accuracy, while the maximum number of solver steps is set to $10^{5}$.

Optimization runs may terminate in one of three ways: by ODE solver failure, by fulfilling the optimizer’s local convergence criterion, or by reaching the optimizer’s iteration limits without local convergence. As performance metrics of the optimization, we consider the numbers of failed and locally converged optimization runs.

Additionally, we report the frequency at which the global optimum has been found by the multistart approach. This can be seen as a measure for the complexity of the optimization problem. We define it by counting the number of optimization runs with final objective function value −2 log(L) within a tolerance of $10^{-2}$ from the best observed value.

### 2.11 Local identifiability analysis

To assess the information content of the data sets, we examine which parameters are structurally or practically nonidentifiable. For this purpose, we apply the Identifiability Test by Radial Penalization (ITRP) (Kreutz 2018), a method that detects flat directions in the likelihood function through penalized local optimization. When such a direction is found, the most strongly aligned parameter is classified as nonidentifiable and excluded from further searches. The method can be applied repeatedly until all nonidentifiable parameters are found.

The ITRP algorithm requires initialization at a local optimum. Therefore, we performed local optimization as described above for all modeling problems. For synthetic problems, we use the ground-truth parameters as an initial parameter vector. Although the template problems are already optimized, we run the optimization algorithm to test the ODE solver and optimizer configuration.

The test is then applied with its default configuration: a penalty radius of 1, five radial directions tested per iteration, and a flatness threshold of $10^{-3}$. The maximum number of ODE solver steps was set to $10^{6}$. All other settings are consistent with the multistart parameter estimation described above.

The statistical models have different kinds of parameters: the dynamic parameters (e.g. rate constants and initial values) determine the dynamics of the ODE system, while all remaining parameters are responsible for the mapping from the ODE system to the data by observable and error models. For our problem-generating algorithm, this distinction is especially important, since the ODE system and all dynamic parameters are taken directly from the template problem, while the observable and error parameters are introduced by generation and condition-assignment of the observables. Thus, we define the identifiability ratio for three parameter sets: “all parameters,” the “dynamic parameters,” and the remaining “nondynamic parameters.”

### 2.12 Principal component analysis

To visualize the structure of the benchmark collection, we perform a PCA on 14 numerical features that capture key aspects of problem size, experimental design, parameter identifiability, and optimization behavior. The selected features are:

- Template characteristics: number of dynamic variables, estimated dynamic parameters, TC experiments, and DR experiments.
- Problem characteristics (see Section 2.4.1): number of observables, estimated parameters, and data points; median relative error and measurement time.
- Parameter estimation (see Section 2.4.2): fractions of failed and converged runs, and frequency of recovering the global optimum (best observed optimum).
- Parameter identifiability (see Section 2.4.3): identifiability ratios for dynamic and nondynamic parameters.

Note that the first four features are defined by the template problem alone and are therefore shared across all corresponding synthetic problems. The remaining features vary at the level of individual benchmark problems.

To ensure numerical stability and comparability, all count-based features *x* are log-transformed using ${\;\text{log}\;}_{10}(x+10^{-6})$ and then standardized. Ratio-based metrics are standardized directly without transformation.

The PCA is conducted on the full benchmark set, including both template and synthetic problems. Benchmark problems with missing values—e.g. due to failed identifiability analysis—need to be excluded. Importantly, exclusion of a template problem does not necessarily imply exclusion of derived synthetic problems.

## 3 Results

### 3.1 Exemplary comparison: template versus synthetic

To illustrate the similarity between real and synthetic problems, we compare the template problem “Bachmann” (Bachmann et al. 2011) with one of its derived synthetic problems, “Bachmann_RS47.” The template problem comprises 541 data points for 10 observables and 115 estimated parameters, while the synthetic problem includes 492 data points for 8 observables and 69 parameters.

Figure 1 provides a side-by-side comparison of the two problems. The experiment-observables matrices in the left columns (panels A and D) display similar measurement patterns across experiments. This showcases that most columns of the synthetic EOM are sampled from the template EOM without modification.

We emphasize that the columns in the template and synthetic EOM do not correspond to the same observable expressions. In the middle column (panels B and E), we present two exemplary observable definitions for each problem. In contrast to the template, the synthetic observables do not include any transformations or unknown parameters. Still, both problems contain both individually observed dynamic variables, as well as compound measurements of similar complexity. Thus, the synthetic observable complexity seems comparable to real-world models.

The last column (panels C and F) shows selected TC and DR data sets for these observables. The plots cannot be compared directly due to different observable definitions and randomly perturbed dynamic parameters of the synthetic problem. Nonetheless, the synthetic data are similar to the template data in key qualitative features: the number of data points per experiment, the sampling density, and the relative noise levels.

### 3.2 Data and model characteristics

To assess the realism and diversity of the generated benchmark problems, we computed five key characteristics: number of observables, number of parameters, number of data points, median relative error, and median measurement time.

Looking at the collection as a whole, the median synthetic problem contains 9 dynamic variables, 4 observables, 106 data points, and 37 estimated parameters—along with a median relative error of 13% and a median measurement time of 74 s (see Fig. 2). These values fall well within the range of realistic calibration scenarios. The pooled synthetic distributions, visualized by stylized boxplots in the panel backgrounds, are symmetrical, contain few outliers, and span ranges similar to those of the template problems. This supports the realism and plausibility of the synthetic modeling problems.

**Figure 2:**
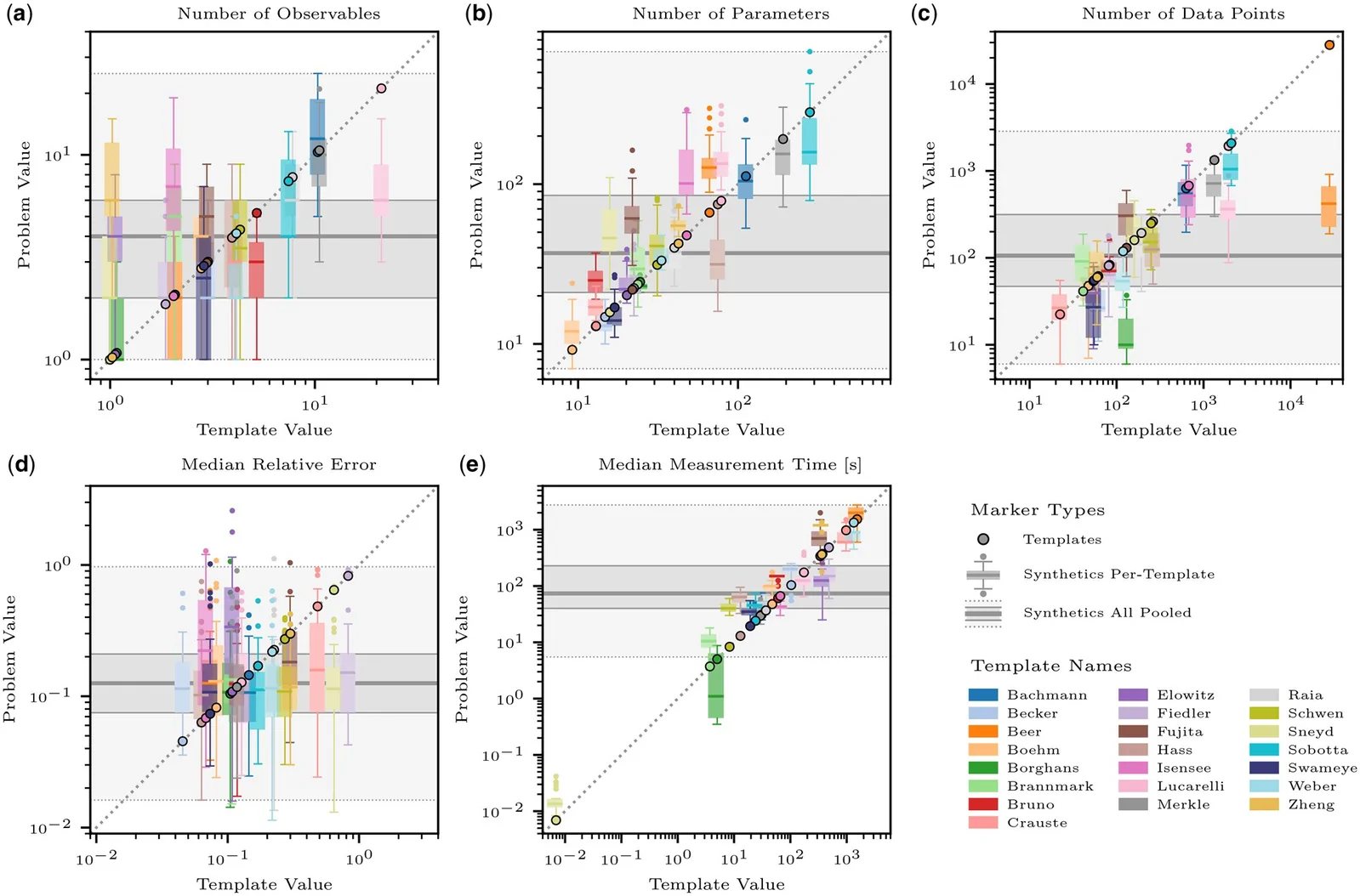
Comparison of synthetic and template problem characteristics. Each panel shows the values for the template problem (horizontal axis) versus the distributions across its 50 synthetic counterparts (vertical axis) for (a) number of observables, (b) number of parameters, (c) number of data points, (d) median relative error, and (e) median measurement time. Small markers show outliers of the per-template distributions. Colors indicate the template name (e.g. Bachmann (Bachmann *et al.* 2011), Becker (Becker *et al.* 2010)). Slight horizontal jitter improves readability. The background shows pooled distributions across all synthetic problems as gray boxplots: solid lines for median and quartiles, dotted lines for whiskers (1.5× IQR), and shaded areas for visual guidance.

Stratified per-template distributions are generally symmetrical with few outliers and exhibit substantial internal variability. As a result, the distributions of different templates frequently overlap. This indicates that the synthetic problems do not form isolated clusters around their respective templates but instead constitute a unified collection that spans a broad and continuous problem space.

The collection of synthetic problems also includes combinations of model characteristics not observed in the original template set. This indicates that our algorithm substantially enhances the problem diversity.

### 3.3 Multistart parameter estimation

To illustrate a typical modeling task, we performed multistart parameter estimation on all synthetic and template problems. For each problem, 100 optimization runs were conducted with randomly initialized parameter values. Figure 3 shows the distributions of three key metrics: the number of failed runs, locally converged runs, and best observed optimum hits. In addition to boxplots, individual values are shown as strip plots to highlight the difference in group sizes between synthetic and template problems. Although template and synthetic problems show substantial overlap, the synthetic collection contains additional challenging cases in the tails of the distributions, including problems with high optimization failure rates and low recovery rates of the best observed objective value.

**Figure 3:**
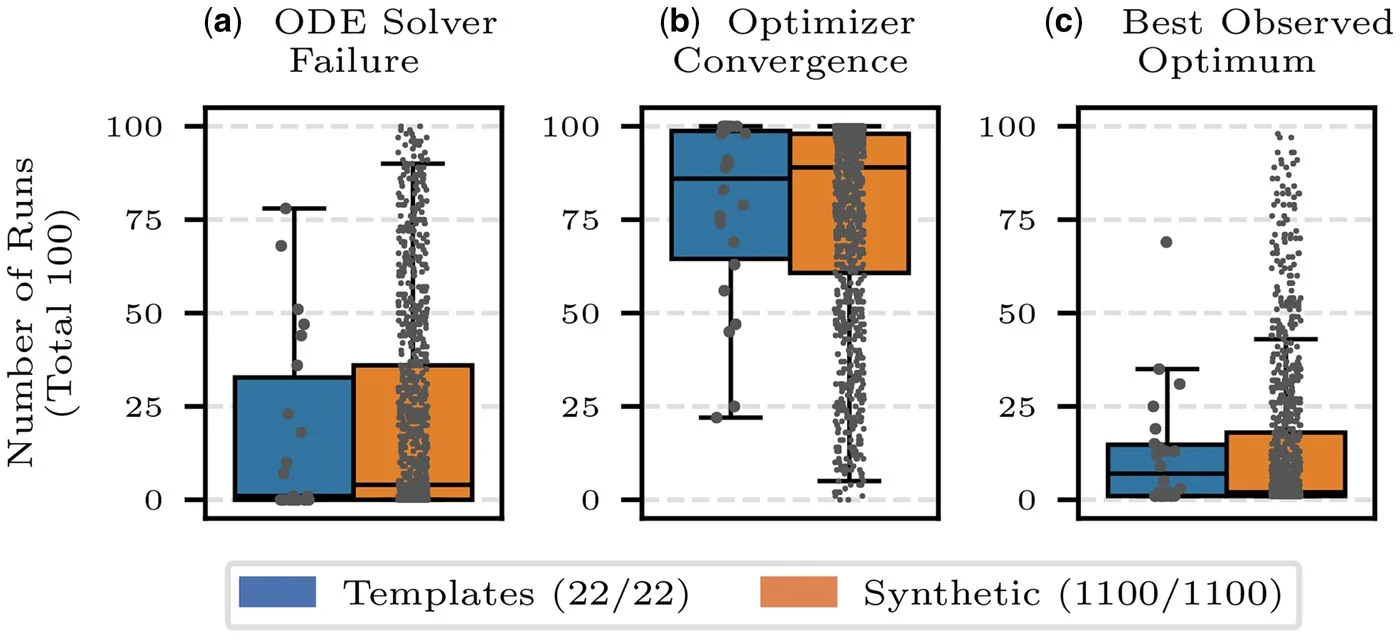
Counting statistics for multistart parameter estimation. (a) Number of optimization runs with ODE solver failure. (b) Number of runs reaching the local convergence criterion. (c) Number of runs that recover the best objective value. In each panel, the left boxplots show results for template problems, and the right boxplots for synthetic problems. Individual values are overlaid as strip plots. The legend indicates that all problems were included in the analysis. Synthetic benchmarks exhibit substantial optimization diversity, while the majority can be optimized sufficiently well.

Most synthetic problems are reasonably well optimizable. Specifically, 75% of problems had fewer than 36 failures and more than 60 converged runs out of 100. These thresholds correspond to the boxplot quartiles shown in panels A and B. For the template problems, we observed similar performance, with 75% of problems below 33 failures and above 64 converged runs. Notably, the synthetic distribution has a longer tail toward “hard-to-optimize” cases, where the majority of runs fail due to ODE solver errors. This reflects that the generation process not only replicates typical optimization challenges but also produces more varied—and sometimes less polished—modeling problems.

To assess the complexity of the optimization landscape, we also counted how often the global optimum was recovered—defined as reaching the best observed objective value within a tolerance of $10^{-2}$. For 43% of synthetic and 31% of template problems, the best value was found only once, suggesting potentially complex optimization landscapes or numerical instabilities. Due to the large number of synthetic problems, the distribution again shows a long tail, this time toward “easier” problems with high recovery rates of the best observed optimum. However, the upper quartile remains low (18/100 for synthetic and 15/100 for template problems), indicating that most problems exhibit non-trivial optimization landscapes. Objective-value gap analyses indicate that many benchmark problems repeatedly yield solutions close to the best observed objective value, while lower-ranked runs reveal increasing heterogeneity among optimization outcomes (Supplementary Fig. S1). Recurrence analyses of terminal objective-value levels show that many detected levels are isolated outcomes, whereas only a limited number recur consistently across optimization runs (Supplementary Fig. S2). In summary, the synthetic problems reflect a realistic spectrum of optimization behavior, including both well-behaved and challenging cases. This confirms that the benchmark set achieves its goal of combining realism with diversity in optimization difficulty.

### 3.4 Local identifiability analysis

To evaluate the information content of the benchmark problems, we analyzed parameter identifiability using the ITRP (Kreutz 2018). This method searches for flat directions in the likelihood landscape to identify structurally or practically nonidentifiable parameters.

The test could be successfully performed for 21 of the 22 template problems and 993 of the 1100 synthetic problems. Most failures stemmed from ODE solver issues during local optimization, likely due to unadjusted solver settings that were kept fixed for comparability.

Figure 4 shows the distribution of identifiable parameter ratios. When considering all parameters (panel A), template and synthetic problems overlap substantially, but synthetic problems include additional cases with reduced identifiability and have a slightly lower median identifiability ratio (87% versus 93%).

**Figure 4:**
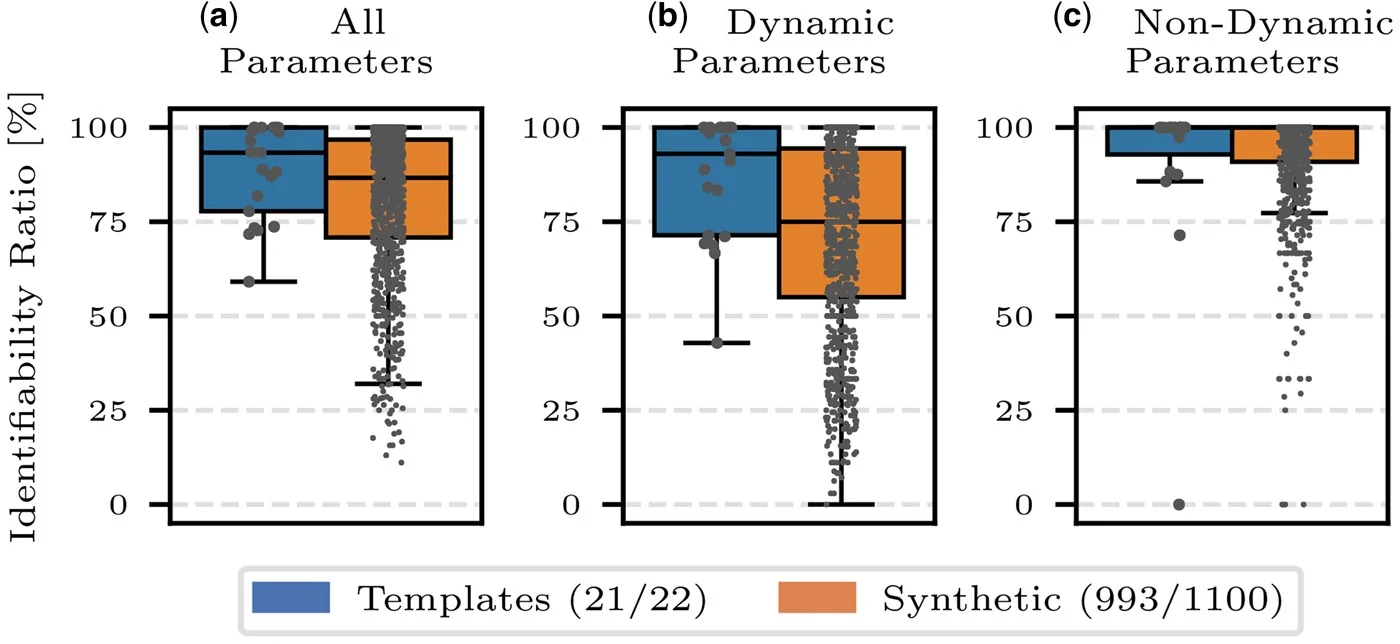
Ratio of identifiable parameters for (a) all parameters, (b) dynamic parameters, and (c) non-dynamic parameters. In each panel, the left boxplots show results for template problems, and the right boxplots for synthetic problems. Individual values are overlaid as strip plots. The legend indicates the number of problems with successful analysis results. Synthetic problems include additional cases with reduced identifiability, especially among dynamic parameters.

Separation in parameter subsets reveals that this difference clearly arises from the dynamic parameters (panel B). Here, synthetic problems have a wider distribution, with quartiles ranging from 55% to 94%, whereas template problems range from 71% to 100%. In contrast, nondynamic parameters (panel C) are consistently well identifiable in both groups, with both medians at 100% and lower quartiles at 91% for synthetic and 93% for template problems.

These results suggest that reduced identifiability in synthetic problems is primarily due to limited observability of dynamic system components. This is consistent with the randomized construction of observation functions, which may leave some states insufficiently constrained.

### 3.5 Analysis of observation-function and EOM characteristics

To investigate how our simulation results are associated with the assumptions we make about the structure of generated observables and EOMs, we analyzed the sensitivity of the previous outcomes to such assumptions. Complete methodological details are provided in the supplementary material. Within each template group, we utilized the natural variation in (i) ratio of scale and offset parameters, (ii) ratio of summed observables, (iii) number of observables, (iv) mean pairwise Jaccard similarity between observable columns, and (v) standard deviation of the pairwise Jaccard similarities to investigate how parameter-estimation and identifiability outcomes depend on these simulation characteristics.

Higher ratios of scale and offset parameters and summed observables, as well as smaller numbers of observables, were interpreted as greater observation-function complexity. Across templates, greater observation-function complexity was generally associated with lower identifiability and poorer multistart parameter-estimation outcomes (Supplementary Table S2). This relationship was more consistent for the ratio of scale and offset parameters and the number of observables than for the ratio of summed observables. Dynamic-parameter identifiability and best observed objective recovery are shown as representative examples in Supplementary Fig. S3.

Observable co-occurrence in the EOMs, summarized by the mean and standard deviation of pairwise Jaccard similarities, showed no consistent relationship with the investigated outcomes (Supplementary Table S3).

### 3.6 PCA projection of problem space

To complement the detailed evaluation metrics, we performed a PCA to obtain a low-dimensional representation of the benchmark collection. Of the 1122 problems, 1010 (19 templates and 991 synthetics) had complete data for all 14 selected characteristics (see Section 2.4.4) and were included in the analysis. Note that exclusion of a template problem does not imply exclusion of the derived synthetic problems.

Figure 5 shows a scatterplot of the first two principal components, which together explain 49.8% of the total variance. Many synthetic problems are located close to their corresponding templates, indicating successful preservation of core characteristics. At the same time, the synthetic problems exhibit greater variability, leading to substantial overlap between template-specific regions and filling in much of the space between them. As a result, the synthetic collection forms a nearly continuous distribution, which only includes two larger gaps between otherwise connected clusters.

**Figure 5:**
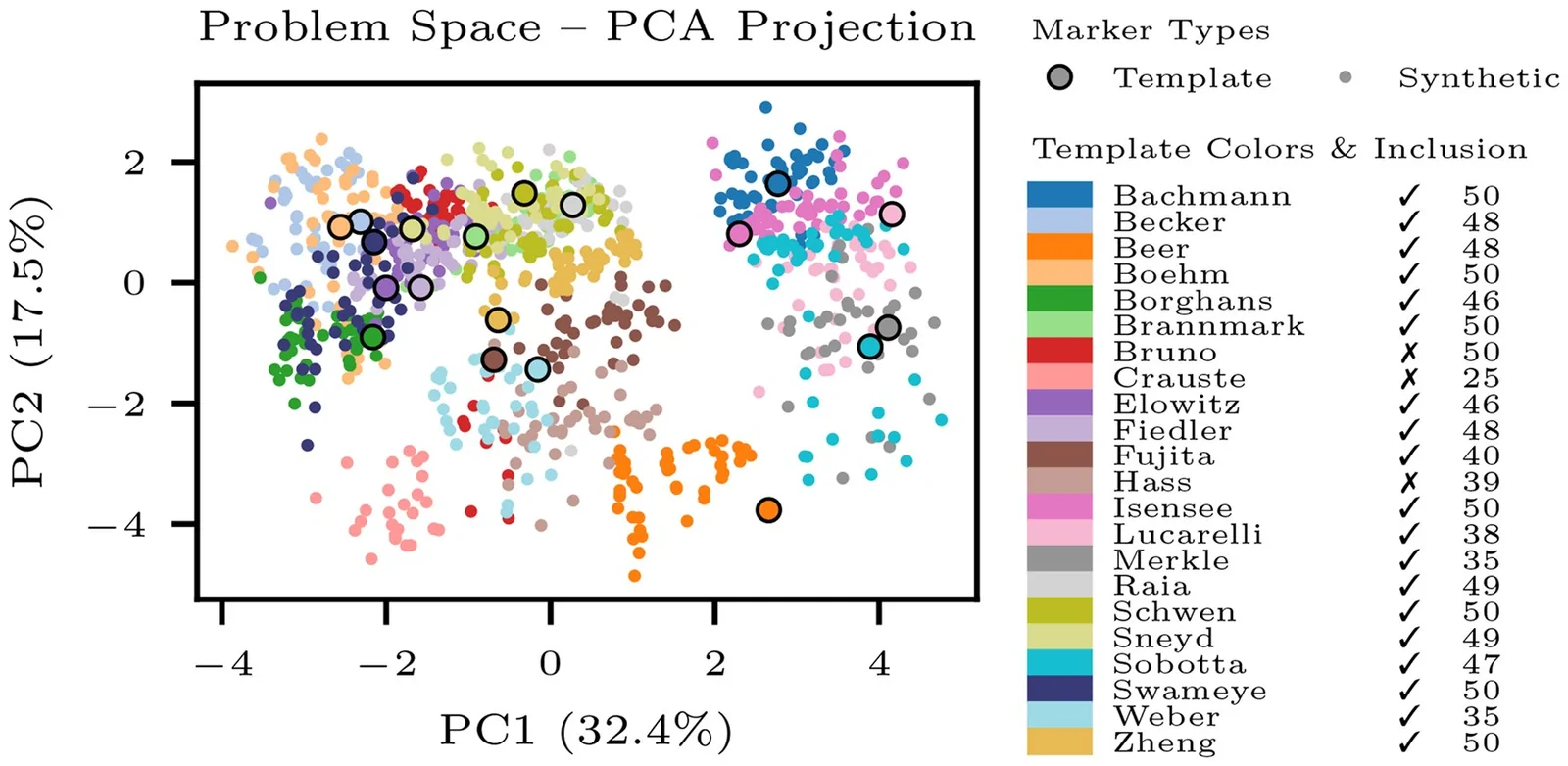
PCA projection of the problem space based on 14 problem characteristics. Template problems are shown as edge-highlighted circles and synthetic problems as small dots; colors indicate the corresponding template. Problems with incomplete feature vectors are excluded form the PCA. The legend reports for each template, its inclusion status (✓ or ✗), and the number of included synthetic problems.

## 4 Discussion

In this work, we introduced a large-scale collection of 1100 synthetic benchmark problems for dynamic modeling of cellular processes. Each problem is based on one of 22 template modeling problems that provide the biological context and modeling structure in the form of ODE equations, definitions of experimental conditions, and their relationship to measured observables. Our algorithm generates synthetic datasets for randomly sampled observables with realistic measurement patterns across experimental conditions. This results in novel synthetic modeling problems. Both the synthetic benchmark collection and the problem-generation algorithm are made publicly available to the scientific community.

### 4.1 Realism and diversity

A central strength of our approach is combining the realism of experimental models with the scalability of synthetic data generation. The two key evaluation criteria for the resulting benchmark collection are realism and diversity: Are the synthetic problems representative of real-world modeling tasks? And do they go beyond the templates to add value?

To evaluate these aspects, we analyzed the benchmark problems in terms of size, complexity, noise level, optimization performance, and parameter identifiability (Kreutz 2018).

The realism of the synthetic problems is supported by multiple observations. The underlying ODE systems and experimental designs are based on experimentally derived biological models. The synthetic summary statistics (e.g. number of data points, parameters, noise levels) fall into realistic ranges and typically encompass the template values. Moreover, many synthetic problems exhibit similar optimization performance and parameter identifiability.

Beyond realism, the benchmark collection demonstrates substantial diversity. The synthetic distributions are continuous and wide, often exceeding the ranges of the templates. The multistart optimization results and identifiability tests reveal that the synthetic problems cover a wide spectrum—from simple, well-constrained models to highly challenging or partially unidentifiable ones. While the latter may initially appear unrealistic, these problems can be interpreted to reflect common intermediate stages in real-world modeling workflows, where model refinement (e.g. in terms of model selection or reduction) is still ongoing. Numerical failures and heterogeneous optimization or identifiability outcomes are therefore an expected part of this benchmark spectrum and provide information about the robustness of computational methods under realistic modeling difficulties.

Together, these observations show that the synthetic benchmarks not only replicate known problem types but also enrich the problem space by including new combinations of characteristics and varying degrees of complexity.

### 4.2 Applications

The benchmark collection is intended as a general-purpose resource for evaluating different computational and conceptual modeling methods in systems biology. Existing method-comparison studies, for example on parameter optimization approaches (Villaverde *et al.* 2019) or prediction uncertainty quantification philosophies (Villaverde *et al.* 2023), are often conducted only on a limited set of case studies of systems biology models, which our large collection can help overcome in future method-comparison studies.

Our collection addresses another key limitation of such case-study analyses: published models portray a clean model state, in which the available data are well tailored to the model complexity and parameter uncertainties are already decently constrained. Due to the randomized experimental designs with only partial observations, our benchmark models are less numerically stable and include more nonidentifiable parameters, representing more intermediate modeling stages that can also be particularly useful for testing model selection and reduction methods. Additionally, particularly difficult individual problems or subsets can serve as test cases in the development of new computational methods.

Thus, our benchmark set allows for more rigorous performance comparisons of parameter estimation approaches, structural and practical identifiability analysis methods, model selection techniques, or uncertainty quantification for model parameters or predictions.

### 4.3 Limitations and outlook

While the template-based simulation approach ensures high realism, it also introduces a nested structure in the synthetic benchmark collection when multiple synthetic problems are generated from the same template problem. Our analysis of general problem characteristics and the PCA projection of the problem space showed a large variability of the generated problems. While there are clusters remaining, we mostly see interconnected and overlapping per-template distributions, which support a reduced impact of the nesting. Nevertheless, this structure is inherent to the synthetic benchmark collection and should always be considered when analyzing benchmark results.

Our collection takes on the ambitious task of simulating a realistic structure of chosen experimental conditions and conducted measurements. This requires many simulation choices, which we made based on what we considered the most realistic scenario feasible for automated problem generation. As other combinations of drawing random observables and specifying experimental conditions might have been chosen to simulate such a benchmark, we can only suggest that our approach does so realistically although we cannot conclusively prove this. Thus, although our collection can safely be used to compare computational modeling methods, it should not be used to infer general properties of systems biology models.

In real-world model development and calibration, the choices of which experiments should be conducted are driven by biological reasoning, feasibility of measurements, and identification of gaps at intermediate model calibration stages. The emerging experimental designs form the characteristics of the optimization problem to a high degree. A model’s sensitivity matrix will typically form both horizontal and vertical block structures, which emerge from correlations of sensitivities within time courses measured for different observables but also from observables that are sensitive to different sets of parameters; the degree of such block structures critically shapes the optimization landscape in terms of its sloppiness of eigenvalues (Tönsing *et al.* 2014). With sparse data, models may furthermore exhibit multiple alternative good-fitting solutions or candidate local minima along poorly constrained directions, which can be reduced to a single well-constrained fit by adding informative experiments (Transtrum and Qiu 2012). An especially important aspect of informative experimental design is complementarity, i.e. selecting experiments that probe parameter-sensitive directions or effective degrees of freedom not already constrained by the available data (Apgar *et al.* 2010, Transtrum and Qiu 2012). While we keep the collection as realistic as possible by virtue of the template problems they are based on, our approach cannot faithfully represent the intentionality of the experimental design decisions that drive real-world model development and calibration.

In the future, the variability of generated problems could be further increased by extending the problem-generating algorithm. For instance, the definitions of experimental conditions could be generated synthetically, or the ODE equations could be randomly manipulated (e.g. by adding or removing reactions). However, such structural modifications would require additional criteria to ensure that the generated ODE systems remain biologically interpretable and consistent with plausible reaction mechanisms. Moreover, the problem-generating algorithm could be modified to give the user control over certain problem characteristics. This way, one could intentionally generate specific synthetic problems instead of relying on randomization results.

## 5 Conclusion

We introduced a large collection of 1100 realistic and diverse synthetic benchmark problems for dynamic modeling. By densely populating and extending the problem space defined by published models, the collection serves as a valuable resource for systematic method development, evaluation, and benchmarking in systems biology.

## Supplementary Material

vbag307_Supplementary_Data

## Data Availability

All modeling problems (template and synthetic), the problem-generation algorithm and all analysis scripts are available on GitHub at https://doi.org/10.5281/zenodo.23211070. Modeling problems are shared in their native Data2Dynamics file format (Raue *et al.* 2015, 2013). We also provide them as PEtab (Schmiester *et al.* 2021) files. But not all problems can be correctly represented in this format. More details are given in the Supplementary material.
