# Supplementary material for: 1100 synthetic benchmark problems for dynamic modeling of cellular processes
Source: Bioinform Adv. 2026 Oct 10;6(1):vbag307. doi: 10.1093/bioadv/vbag307 (PMC13712306; doi:10.1093/bioadv/vbag307)
Supplement: Supplementary file 1 [file vbag307_supplementary_data.zip › S3_ObservationFunctionComplexity.pdf]

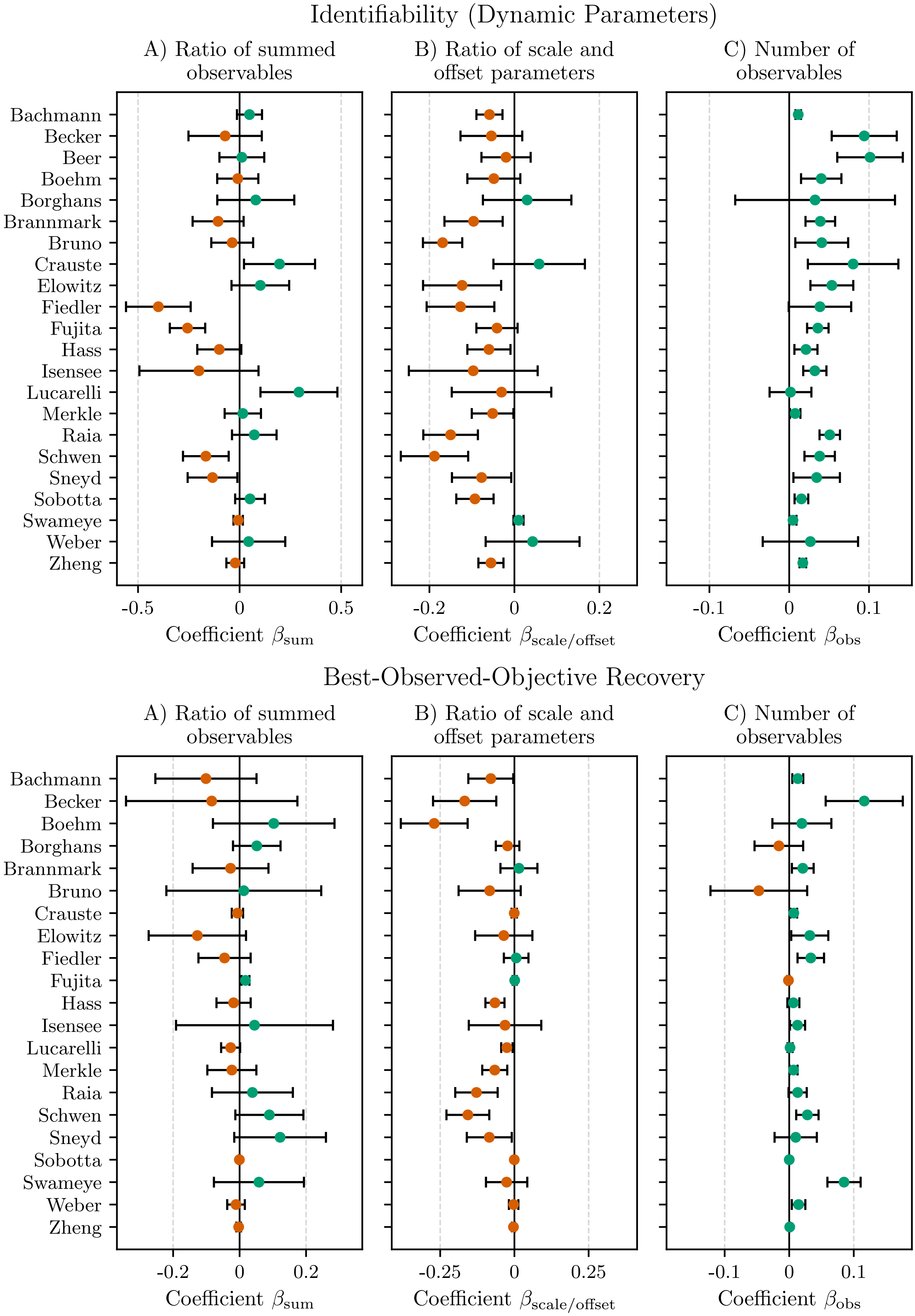

Identifiability (Dynamic Parameters)
A) Ratio of summed observables
B) Ratio of scale and offset parameters
C) Number of observables
Bachmann
Becker
Beer
Boehm
Borghans
Brannmark
Bruno
Crauste
Elowitz
Fiedler
Fujita
Hass
Isensee
Lucarelli
Merkle
Raia
Schwen
Sneyd
Sobotta
Swameye
Weber
Zheng
-0.5
0
0.5
-0.2
0
0.2
-0.1
0
0.1
Coefficient β_{sum}
Coefficient $\beta_{\text{scale/offset}}$
Coefficient β_{obs}
Best-Observed-Objective Recovery
A) Ratio of summed observables
B) Ratio of scale and offset parameters
C) Number of observables
Bachmann
Becker
Boehm
Borghans
Brannmark
Bruno
Crauste
Elowitz
Fiedler
Fujita
Hass
Isensee
Lucarelli
Merkle
Raia
Schwen
Sneyd
Sobotta
Swameye
Weber
Zheng
-0.2
0
0.2
-0.25
0
0.25
-0.1
0
0.1
Coefficient β_{sum}
Coefficient $\beta_{\text{scale/offset}}$
Coefficient β_{obs}
