# Supplementary material for: 1100 synthetic benchmark problems for dynamic modeling of cellular processes
Source: Bioinform Adv. 2026 Oct 10;6(1):vbag307. doi: 10.1093/bioadv/vbag307 (PMC13712306; doi:10.1093/bioadv/vbag307)
Supplement: Supplementary file 1 [file vbag307_supplementary_data.zip › S2_StripBoxPlots_Multistart_Recurrency.pdf]

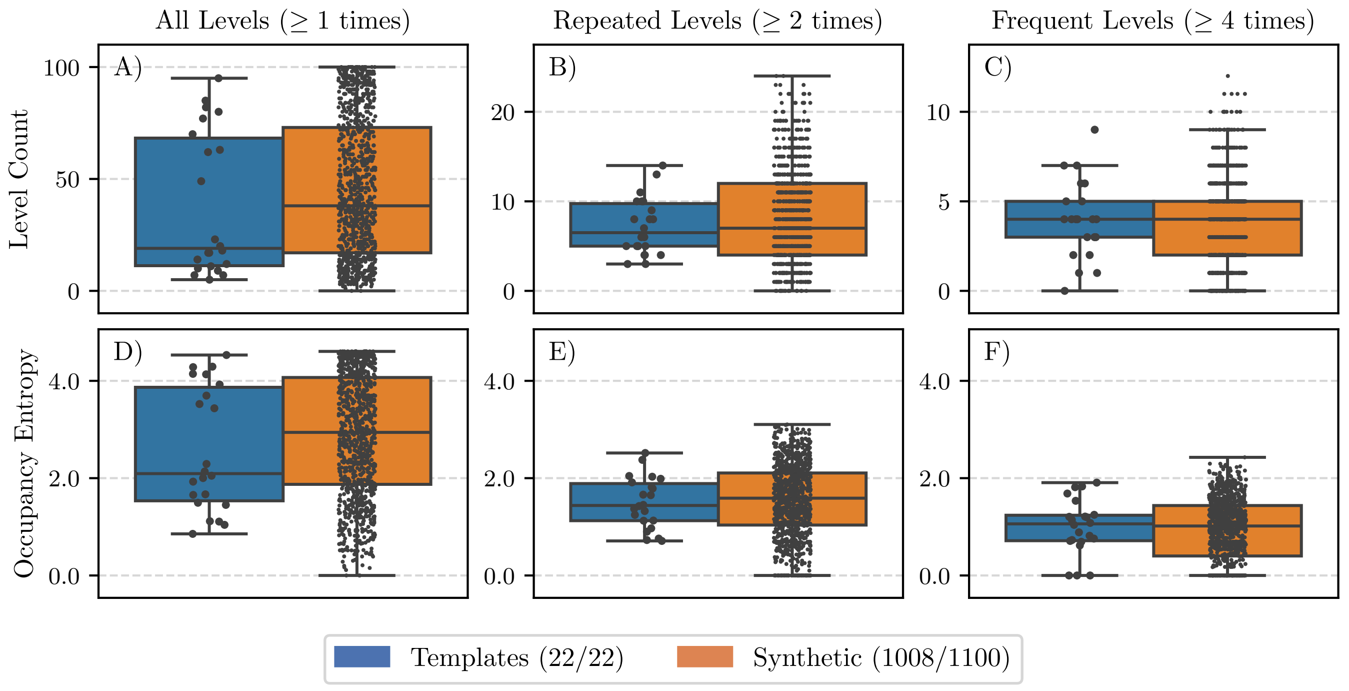
All Levels (≥ 1 times)
Repeated Levels (≥ 2 times)
Frequent Levels (≥ 4 times)
Level Count
Occupancy Entropy
A)
B)
C)
D)
E)
F)
100
50
0
20
10
0
10
5
0
4.0
2.0
0.0
Templates (22/22)
Synthetic (1008/1100)
