# Supplementary material for: 1100 synthetic benchmark problems for dynamic modeling of cellular processes
Source: Bioinform Adv. 2026 Oct 10;6(1):vbag307. doi: 10.1093/bioadv/vbag307 (PMC13712306; doi:10.1093/bioadv/vbag307)
Supplement: Supplementary file 1 [file vbag307_supplementary_data.zip › S1_StripBoxPlots_Multistart_Fit-Differences_Extended.pdf]

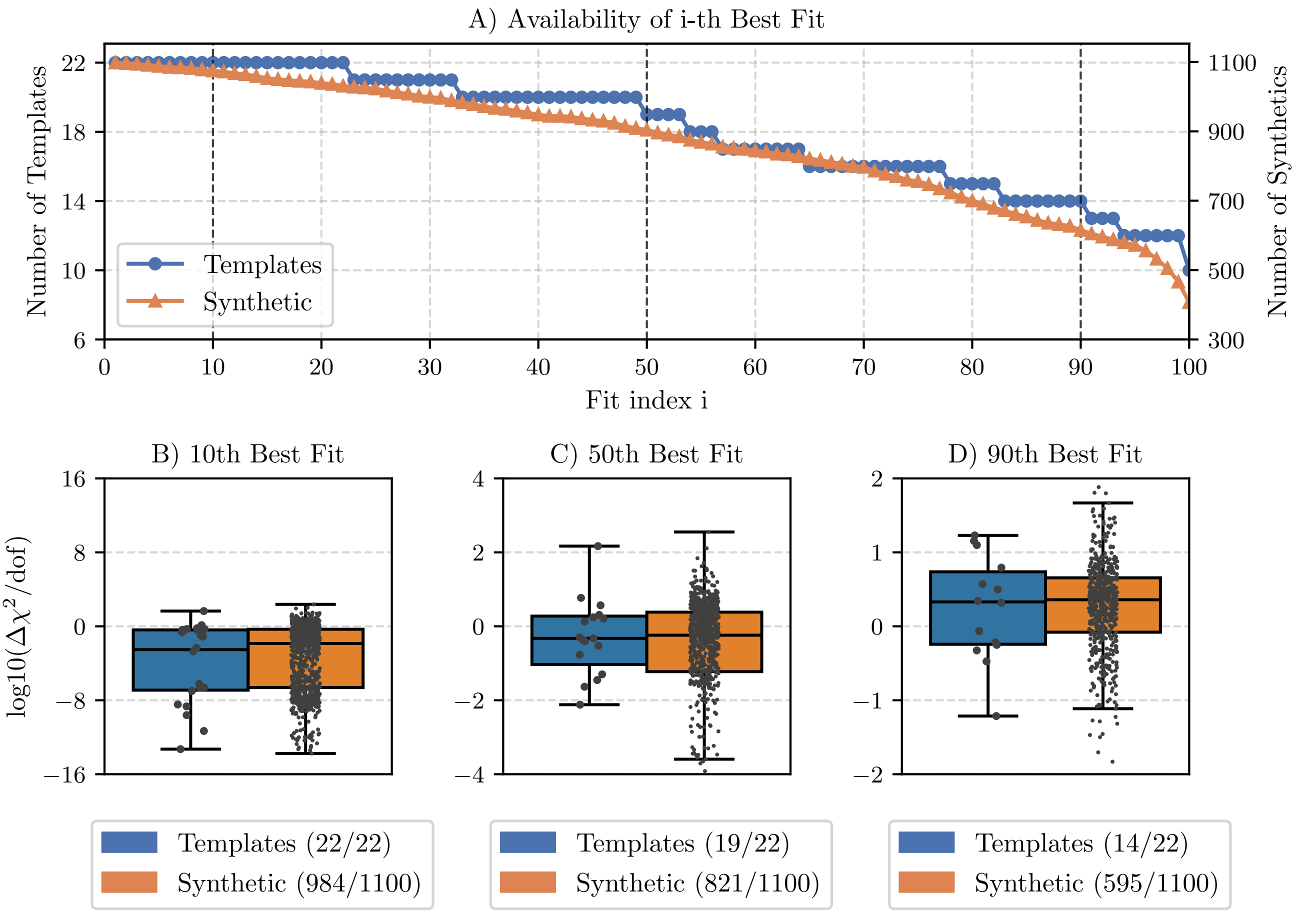

A) Availability of i-th Best Fit
Number of Templates
Number of Synthetics
Fit index i
Templates
Synthetic
B) 10th Best Fit
C) 50th Best Fit
D) 90th Best Fit
log10(Δχ²/dof)
Templates (22/22)
Synthetic (984/1100)
Templates (19/22)
Synthetic (821/1100)
Templates (14/22)
Synthetic (595/1100)
