# Supplementary material for: 1100 synthetic benchmark problems for dynamic modeling of cellular processes
Source: Bioinform Adv. 2026 Oct 10;6(1):vbag307. doi: 10.1093/bioadv/vbag307 (PMC13712306; doi:10.1093/bioadv/vbag307)
Supplement: Supplementary file 1 [file vbag307_supplementary_data.zip › SupplementaryMaterial.pdf]

1100 Synthetic Benchmark Problems for Dynamic Modeling of Cellular Processes

Supplementary Material

Niklas Neubrand[1,†,*], Timo Rachel[1,†], Tim Litwin[1,2], Jens Timmer[2,3,4], Clemens Kreutz[1], Moritz Hess[1]

1 Institute of Medical Biometry and Statistics, Faculty of Medicine and Medical Center, University of Freiburg, Germany
2 Center for Data Analysis, Modeling and AI (FDMAI), University of Freiburg, Germany
3 Institute of Physics, University of Freiburg, Germany
4 Centre for Integrative Biological Signalling Studies (CIBSS), University of Freiburg, Germany

† These authors contributed equally to this work.
*** corresponding author:** neubrand.niklas@gmail.com

1 Experimental Template Problems

1.1 Template Overview

Table S1 lists all templates together with their problem characteristics and the original references.

Template	States	Experiments TC	DR	Observ-ables	Parameters Dyn	All	Data Points	Median Error [%]	Median Time	Reference
Bachmann	25	6	4	10	29	115	541	15	30 s	(Bachmann et al., 2011)
Becker	6	1	0	3	9	14	72	5	2 min	(Becker et al., 2010)
Beer	4	19	0	2	70	72	27132	8	30 min	(Beer et al., 2014)
Boehm	8	1	0	3	6	9	48	9	45 s	(Boehm et al., 2014)
Borghans	3	1	0	1	20	23	111	11	5 s	(Borghans et al., 1997)
Brannmark	9	2	4	2	14	22	43	12	5 s	(Brännmark et al., 2010)
Bruno	7	6	0	5	13	13	77	11	1 min	(Bruno et al., 2016)
Crauste	5	1	0	4	12	12	21	44	13 min	(Crauste et al., 2017)
Elowitz	8	1	0	1	16	19	58	11	5 min	(Elowitz and Leibler, 2000)
Fiedler	6	3	0	2	12	22	72	82	6 min	(Fiedler et al., 2016)
Fujita	9	6	0	3	19	22	144	27	8 min	(Fujita et al., 2010)
Hass	9	4	4	4	0	72	221	7	15 s	(Hass et al., 2017)
Isensee	25	15	16	2	34	46	687	7	1 min	(Isensee et al., 2018)
Lucarelli	33	11	1	21	72	84	1755	12	3 min	(Lucarelli et al., 2018)
Merkle	23	16	11	11	43	199	1137	13	30 s	(Merkle et al., 2016)
Raia	14	4	0	8	18	39	205	25	35 s	(Raia et al., 2011)
Schwen	11	7	0	4	13	30	286	26	8 s	(Schwen et al., 2015)
Sneyd	6	8	0	1	14	15	135	72	8 ms	(Sneyd and Dufour, 2002)
Sobotta	13	32	21	8	23	260	2222	18	20 s	(Sobotta et al., 2017)
Swameye	9	1	0	3	10	17	46	7	15 s	(Swameye et al., 2003)
Weber	7	3	0	4	26	36	135	23	24 min	(Weber et al., 2015)
Zheng	15	1	0	1	45	46	60	29	8 min	(Zheng et al., 2012)

Table S1: Overview of all experimental template problems, including key model characteristics and original references. "TC" and "DR" refer to time-course and dose-response experiments, respectively. The parameter counts include only those estimated from experimental data, with "Dyn" indicating the subset of dynamic parameters. Median relative errors are reported as percentages. "Median Time" refers to the median measurement times of the data.

1.2 Implementation Details

The exact implementations of all template problems can be found in our GitHub repository at https://github.com/niklasneubra

For most templates, the implementation is directly taken from the "Examples" folder of the Data2Dynamics toolbox (Raue et al., 2013, 2015). However, for some templates we made slight implementation changes due to technical reasons. These changes are described in the following.

1.2.1 Becker

In the modeling problem of Becker et al. (Becker et al., 2010) parameters are estimated jointly for an ODE system for Erythropoetin (Epo) receptor internalization and a Michaelis-Menten model for the binding kinetics. Since our problem generating algorithm currently only supports modeling problems with a single ODE system, we removed the Michaelis-Menten model to create the simulation template.

1.2.2 Merkle

The modeling problem of Merkle et al. (Merkle et al., 2016) was constructed to identify cell-type specific differences in Epo-induced JAK2-STAT5 signaling. It was originally implemented in the Data2Dynamics toolbox by utilizing two very similar ODE systems – each representing one cell type. To align this with our problem generation algorithm, we modified this implementation to use a single ODE system only. The cell-type specific variations of this ODE system are represented by parameter replacements in the definitions of additional experimental conditions. To summarize, we transformed the modeling problem to another equivalent representation, without altering the parameter estimation task.

1.2.3 Swameye

The ODE system of Swameye at al. (Swameye et al., 2003) uses a spline function to model the input of phosphorylated Epo receptors to the cell. While the time-points of the spline knots were predetermined by the authors, the amplitudes are free parameters that need to be estimated from experimental data.

In our problem-generating algorithm all free parameters – including the spline amplitudes – are randomly perturbed. We observed that this randomization induced divergent behavior of the input function for larger simulation times. Therefore, we decided to further constrain the shape of the input spline by adding additional splines with fixed time-points and amplitudes. Specifically, we replace the original 5-knot spline by a 10-knot spline to force the input function towards small values for large simulation times. This way, we keep the original set of dynamic parameters, while allowing some controlled randomization of the input dynamics.

2 Code Implementation Details

2.1 Working with Data2Dynamics

The simulation pipeline is implemented in MATLAB and utilizes the Data2Dynamics toolbox (Raue et al., 2015, 2013).

Template problems can be provided in the Data2Dynamics file formats. Our distinction between time-course and dose-response experiments is based on the model implementation provided by the user. Data definition files (`data.def`) with identifier `PREDICTOR-DOSERESPONSE` are interpreted as a dose-response experiment.

The benchmark problems can easily be loaded with the Data2Dynamics toolbox by navigating to the respective problem folder and running the commands: `arInit; Setup`. Then, any analysis implemented in Data2Dynamics code can be performed.

2.2 Support for PEtab

Template problems can also be provided in the interoperable PEtab format (Schmiester et al., 2021) and then be translated to Data2Dynamics files using the import function `arImportPEtab`.

Since the PEtab format does not include an identifier for dose-response experiments, all simulation conditions are by default interpreted as time-course experiments. This can be changed by manual modifications in the imported Data2Dynamics files.

We also provide the benchmarks in PEtab files for usage outside of the Data2Dynamics toolbox. Unfortunately, the files exported from the Data2Dynamics toolbox use non-standard mathematical expressions in the condition definitions. This problem affects 6 of the 22 template problems and the corresponding derived synthetic benchmarks. With the upcoming release of PEtab v2, these kinds of condition expressions are expected to be officially supported and this limitation could be removed.

2.3 Computational Robustness

The template problems are diverse in computational complexity and modeling features – such as pre-equilibration, input functions, or error parameter estimation. This imposes high requirements for versatility and robustness of our problem-generation algorithm. During its development we improved it iteratively to catch a large portion of typical errors.

With the current version of the algorithm, all 50 problem generation runs terminated successfully for 18 out of 22 template problems. For the remaining 4, occasional numerical issues when solving the underlying ODE systems required restarting the problem-generation algorithm with different random seeds. The most problematic case was the template based on the model by Isensee et al. (Isensee et al., 2018), which required 67 runs to generate 50 valid synthetic problems (success rate of 75%).

In total, 1,136 problem-generation runs were performed to obtain 50 successful runs for each of the 22 templates, resulting in an overall success rate of 97%.

3 Additional Analysis of Multi-Start Parameter Estimation

The analyses presented in the main article characterize multi-start optimization performance through the fractions of ODE solver failures, local optimizer convergence, and recovery of the best observed objective value. To further characterize optimization-landscape complexity, we analyzed the distributions of terminal objective values obtained from repeated optimization runs.

3.1 Methods

Two limitations should be noted. First, the globally optimal objective value is generally unknown for large-scale nonlinear parameter estimation problems. Consequently, all analyses are referenced to the best objective value observed among the 100 optimization runs. Second, the true number and location of local optima cannot be inferred from finite optimization experiments. The metrics introduced below should therefore be interpreted as empirical characterizations of optimization outcomes rather than direct measurements of the underlying objective-function landscape.

To facilitate comparisons across benchmark problems with different model and data-set sizes, objective-values were normalized by the number of degrees of freedom (dof) defined as the difference between the number of data points n_{data} and the number of parameters n_{par}. As a result, we investigated distributions of the dof-normalized objective-value differences

$$\frac{\Delta\chi_i^2}{\text{dof}} = \frac{\chi_i^2 - \chi_{\text{best}}^2}{n_{\text{data}} - n_{\text{par}}}, \tag{1}$$

where χ_{best}^2 and χ_i^2 are the terminal objective-values of the best and i-th best optimization runs of the given benchmark problem.

To characterize the spread of optimization outcomes, we analyzed quantiles of the terminal objective-value distribution obtained from the 100 multi-start optimization runs. Quantile-based measures were preferred over moment-based statistics such as variances because terminal objective-value distributions are frequently skewed and contain optimization failures (see Fig. 3A of the main text). Specifically, we considered the objective-value differences between the best observed solution and the 10th-, 50th-, and 90th-best optimization runs.

To characterize the diversity of optimization outcomes, dof-normalized terminal objective values were additionally grouped into terminal objective-value levels using a nearest-neighbor clustering criterion with threshold 0.01. Because isolated objective-value levels may arise from numerical artifacts, incomplete convergence, or other non-recurring optimization outcomes, we repeated the analysis using minimum occupancy requirements of one, two, and four optimization runs. Increasing the occupancy threshold progressively emphasizes repeatedly observed solution classes.

3.2 Results

3.2.1 Quantile Analysis

Figure S1 summarizes the distributions of normalized objective-value gaps for the 10th-, 50th-, and 90th-best optimization runs.

The availability analysis in Figure S1A shows the number of benchmark problems for which the i-th best optimization result was available, i.e. the corresponding optimization run terminated without an ODE solver failure. As expected, availability decreases steadily with increasing rank, reflecting the accumulation of failed optimization runs. This effect is more pronounced for synthetic benchmark problems, although valid optimization results remain available for a substantial fraction of benchmark problems even at higher ranks.

Among the subset of benchmark problems with available optimization results, the 10th-best run typically achieved objective values very close to the best observed solution (Figure S1B), indicating that high-quality solutions were frequently recovered. Larger objective-value differences were observed for the 50th-best and 90th-best runs (Figures S1C–D), demonstrating increasing heterogeneity among optimization outcomes as lower-ranked optimization runs are considered.

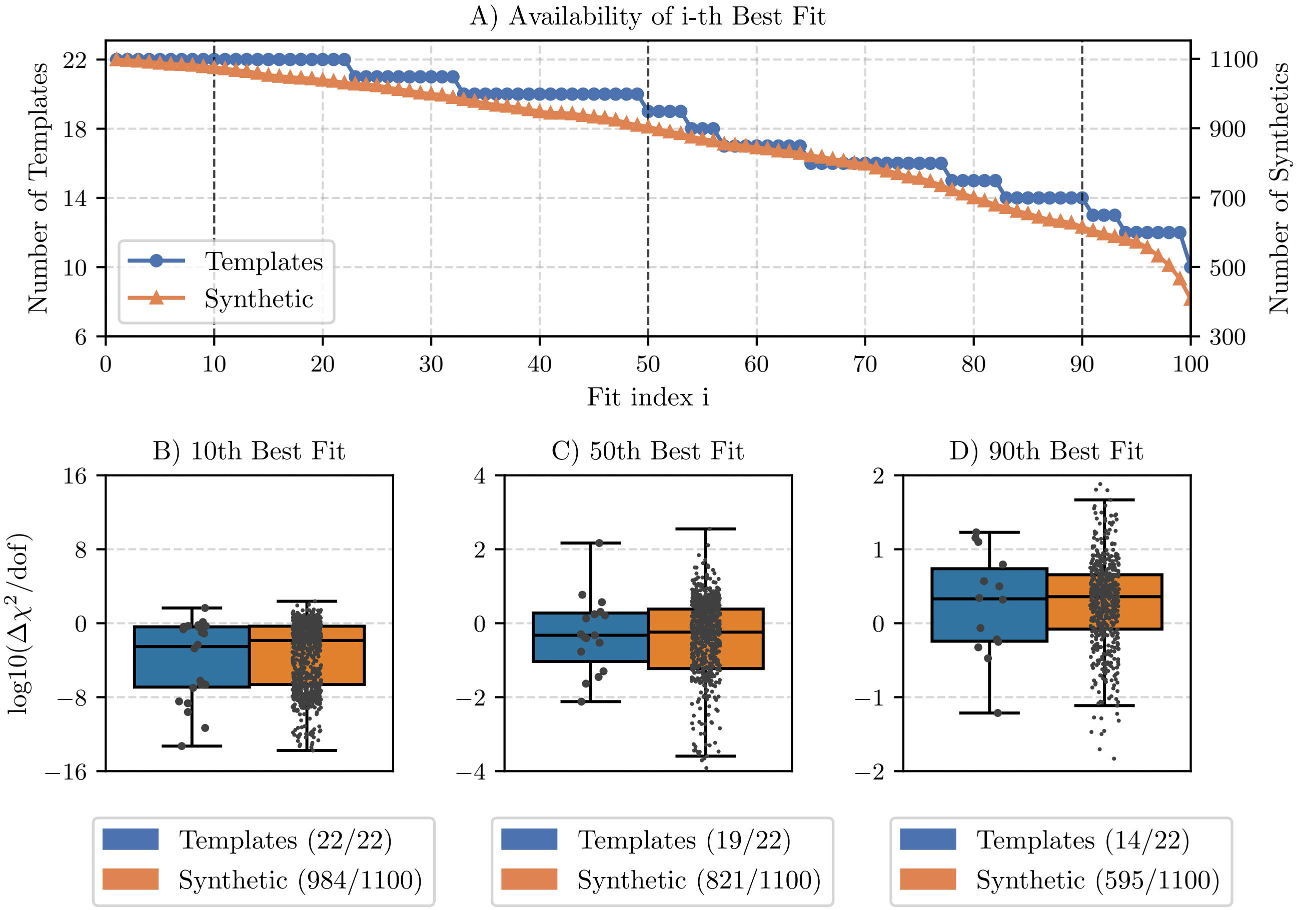

Figure S1: Objective-value differences among multistart optimization runs. Panel A shows the number of benchmark problems for which the i-th best optimization result was available, i.e. the corresponding optimization run terminated without an ODE solver failure. Panels B–D show the distributions of the degrees-of-freedom-normalized objective-value differences between the best observed solution and the 10th-best, 50th-best, and 90th-best optimization run, respectively. Smaller values indicate that a larger fraction of multistart runs converged to objective values close to the best observed solution, whereas larger values indicate greater variability among optimization outcomes. Numbers in parentheses indicate the number of benchmark problems contributing to the respective distributions (see panel A).

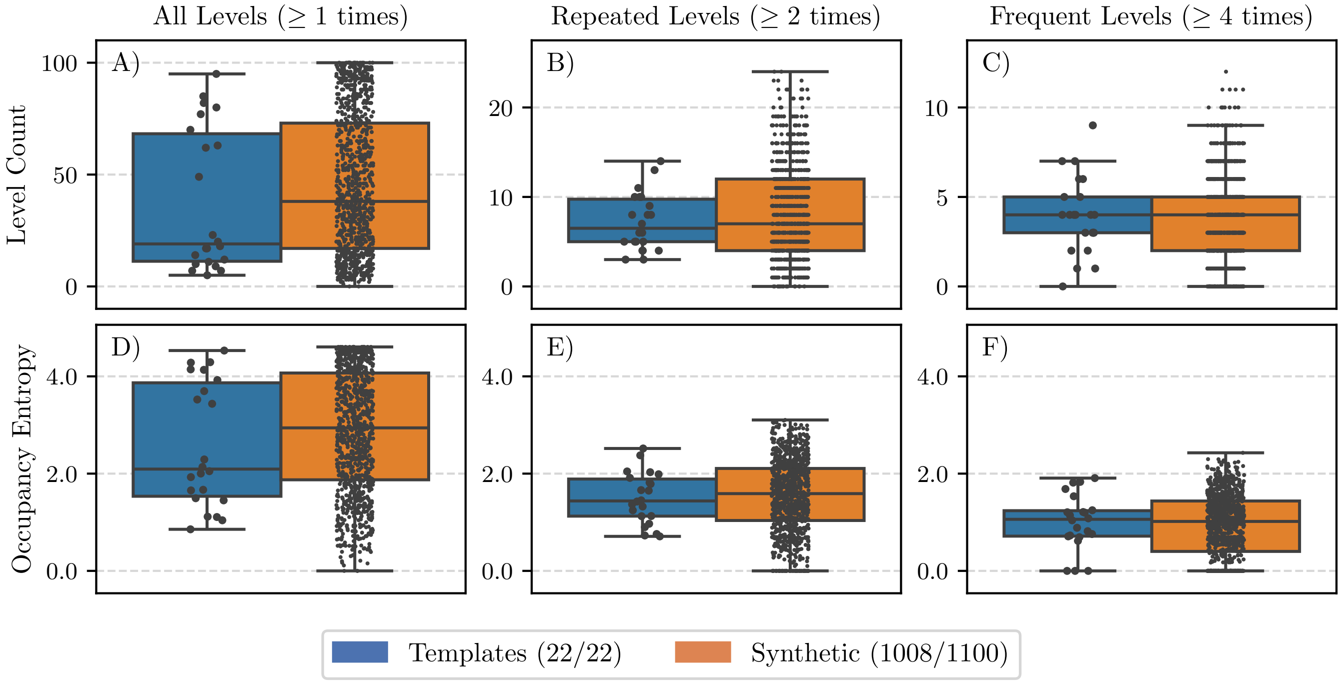

Figure S2: Recurrence of terminal objective-value levels across multi-start optimization runs. Final dof-normalized objective values from 100 optimization runs per benchmark were grouped into terminal objective-value levels using a nearest-neighbor clustering criterion. The top row shows the distribution of the number of detected levels, and the bottom row the corresponding occupancy entropy, which quantifies how evenly optimization runs are distributed among levels. Columns correspond to different minimum occupancy requirements for a level to be counted: all levels (≥ 1 run), repeated levels (≥ 2 runs), and frequent levels (≥ 4 runs). Larger level counts and higher occupancy entropy indicate greater diversity among optimization outcomes. Numbers in parentheses indicate the number of benchmark problems for which at least one level was identified.

Notably, the distributions of normalized objective-value gaps are broadly similar for template and synthetic benchmark problems. Thus, conditional on successful optimization runs being available, both benchmark classes exhibit comparable spreads of terminal objective values. The primary difference between the two benchmark classes therefore appears to arise from the larger number of failed optimization runs among synthetic benchmark problems rather than from substantially different distributions of successful optimization outcomes.

3.2.2 Recurrence Analysis

Figure S2 shows the distributions of recurrent terminal objective-value levels identified using the nearest-neighbor clustering procedure. When all detected levels were counted (≥ 1), many benchmark problems exhibited large numbers of distinct levels, particularly among synthetic benchmark problems. However, requiring levels to be observed repeatedly (≥ 2) substantially reduced the number of detected levels, and a further reduction was observed for frequently recurring levels (≥ 4).

This behavior suggests that a considerable fraction of detected objective-value levels are observed only once and therefore do not represent robustly recurring optimization outcomes. In contrast, the number of repeatedly observed levels remained comparatively small for most benchmark problems, typically ranging from only a few to approximately ten levels.

Increasing the minimum occupancy requirement also reduced the differences in median between template and synthetic benchmark problems. At the same time, the variability of the level counts decreased substantially for template problems. While also present, this reduction of variability is smaller for synthetic problems, suggesting greater heterogeneity of recurring optimization outcomes.

The occupancy entropy analysis revealed a similar pattern. Entropies decreased as stricter occupancy requirements were imposed, indicating that repeatedly observed optimization outcomes tend to concentrate within a limited number of dominant objective-value levels. Differences between template and synthetic benchmark problems were comparatively small with respect to median entropy values. However, synthetic benchmark problems consistently exhibited larger variability, indicating a wider range of recurrence patterns across benchmark instances.

3.3 Discussion

The additional analyses support the conclusions drawn from the main manuscript. Quantile-based objective-value differences demonstrate that many benchmark problems yield high-quality solutions for a substantial fraction of optimization starts, while synthetic benchmark problems exhibit greater variability and slightly reduced robustness. The recurrence analysis further indicates that although numerous terminal objective-value levels can be observed, only a comparatively small number recur consistently across optimization runs. Thus, the empirical complexity of optimization outcomes appears to be driven more by optimization failures and isolated solutions than by a large number of repeatedly observed levels.

4 Analysis of Observation-Function and EOM Characteristics

4.1 Methods

To investigate how observation-function and EOM characteristics relate to the existing multi-start parameter-estimation and identifiability outcomes, we fitted linear regression models separately within each template group.

For the observation-function complexity analysis, the predictors were the ratio of summed observables, the ratio of scale and offset parameters, and the total number of observables. This analysis included all 22 template groups. For each template group and outcome, we fitted the linear regression model

$$y_i = \beta_0 + \beta_{\mathrm{sum}}\, r_{\mathrm{sum},i} + \beta_{\mathrm{scale/offset}}\, r_{\mathrm{scale/offset},i} + \beta_{\mathrm{obs}}\, n_{\mathrm{obs},i} + \varepsilon_i, \tag{2}$$

where i indexes the synthetic benchmark problems within the respective template group. The response y_i denotes one of the investigated multi-start parameter-estimation or identifiability outcomes. The predictor

$$r_{\mathrm{sum},i} = \frac{n_{\mathrm{sum},i}}{n_{\mathrm{obs},i}} \tag{3}$$

denotes the ratio of observables containing a sum to the total number of observables, with each summed observable counted once irrespective of the number of states included in the sum. The predictor

$$r_{\mathrm{scale/offset},i} = \frac{n_{\mathrm{scale},i} + n_{\mathrm{offset},i}}{n_{\mathrm{obs},i}} \tag{4}$$

denotes the ratio of the total number of scale and offset parameters to the total number of observables. An observable containing both a scale and an offset parameter contributes two parameters to the numerator. Finally, $n_{\mathrm{obs},i}$ denotes the total number of observables. Higher ratios of summed observables and scale and offset parameters, as well as smaller numbers of observables, were interpreted as greater observation-function complexity.

While this analysis characterizes the observation functions themselves, the EOM analysis considers how observables are distributed across experiments. For this purpose, observable co-occurrence in each EOM was quantified using pairwise Jaccard similarity. For two observables a and b, it was defined as

$$J_{ab} = \frac{|C_a \cap C_b|}{|C_a \cup C_b|}, \tag{5}$$

where C_a and C_b denote the sets of experiments in which the respective observables were measured. Time-course (TC) and dose-response (DR) experiments were treated jointly when calculating the pairwise Jaccard similarities from the complete EOM. Each benchmark problem was characterized by the mean and standard deviation of the pairwise Jaccard similarities.

An analogous regression model was fitted using these two characteristics as predictors while retaining the number of observables as an adjustment variable. Only observables measured in at least one experiment were included. Individual benchmark problems with fewer than two such observables had undefined Jaccard summaries and were omitted from the corresponding regressions. Separately, entire template groups with only one experiment, identical observable measurement patterns, or otherwise insufficient within-template variation in the pairwise Jaccard summaries were excluded, leaving eight template groups for the EOM analysis.

For both analyses, regression coefficients and corresponding 95% confidence intervals were estimated by ordinary least squares separately for each combination of template group and outcome. Regressions were fitted using complete observations and only when the outcome and all three predictors varied within the respective template group. Both analyses were applied to the identifiability ratios for all, dynamic, and non-dynamic parameters, as well as to the fractions of multi-start runs with ODE solver failures, optimizer convergence, and recovery of the best observed objective value.

4.2 Results

Table S2 and Table S3 summarize the consistency of the coefficient directions across templates for the observation-function and EOM characteristics, respectively.

Table S2: Percentage of positive template-specific regression coefficients for the ratio of summed observables, the ratio of scale and offset parameters, and the number of observables. Values are reported as percentage (n/N), where n is the number of positive coefficients and N the number of fitted template-specific regressions; each coefficient was estimated while adjusting for the other two predictors. Positive coefficients indicate increasing outcome values with increasing predictor values. Greater observation-function complexity corresponds to higher values of the two ratio predictors but a smaller number of observables. For ODE solver failures, $N = 21$ because the outcome did not vary within the Zheng template; for best-observed-objective recovery, $N = 21$ because the outcome did not vary within the Beer template. For all other outcomes, $N = 22$. Overall, greater observation-function complexity was associated with lower identifiability and poorer multi-start parameter-estimation outcomes, with more consistent relationships for the ratio of scale and offset parameters and the number of observables than for the ratio of summed observables.

Outcome	Ratio of summed observables	Scale and offset parameters per observable	Number of observables
Identifiability (all parameters)	50.0% (11/22)	22.7% (5/22)	100.0% (22/22)
Identifiability (dynamic parameters)	45.5% (10/22)	18.2% (4/22)	100.0% (22/22)
Identifiability (non-dynamic parameters)	50.0% (11/22)	0.0% (0/22)	63.6% (14/22)
ODE solver failures	57.1% (12/21)	81.0% (17/21)	38.1% (8/21)
Optimizer convergence	27.3% (6/22)	9.1% (2/22)	72.7% (16/22)
Best-observed-objective recovery	42.9% (9/21)	14.3% (3/21)	85.7% (18/21)

Table S3: Percentage of positive template-specific regression coefficients for mean pairwise Jaccard similarity, the standard deviation of pairwise Jaccard similarities, and the number of observables. Values are reported as percentage (n/N), where n is the number of positive coefficients and N the number of fitted template-specific regressions; each coefficient was estimated while adjusting for the other two predictors. Positive coefficients indicate increasing outcome values with increasing predictor values. For all outcomes, $N = 8$, corresponding to the eight template groups included in the EOM analysis. Overall, neither mean pairwise Jaccard similarity nor the standard deviation of pairwise Jaccard similarities showed a consistent association with the identifiability or multi-start parameter-estimation outcomes across the included template groups.

Outcome	Mean pairwise Jaccard similarity	Pairwise Jaccard standard deviation	Number of observables
Identifiability (all parameters)	75.0% (6/8)	62.5% (5/8)	100.0% (8/8)
Identifiability (dynamic parameters)	62.5% (5/8)	37.5% (3/8)	100.0% (8/8)
Identifiability (non-dynamic parameters)	62.5% (5/8)	50.0% (4/8)	87.5% (7/8)
ODE solver failures	75.0% (6/8)	37.5% (3/8)	25.0% (2/8)
Optimizer convergence	50.0% (4/8)	37.5% (3/8)	75.0% (6/8)
Best-observed-objective recovery	37.5% (3/8)	50.0% (4/8)	100.0% (8/8)

For identifiability, optimizer convergence, and best-observed-objective recovery, negative coefficients for the two observation-function ratio predictors and positive coefficients for the number of observables indicate poorer outcomes with greater observation-function complexity. For ODE solver failures, the corresponding coefficient directions are positive for the ratio predictors and negative for the number of observables.

Overall, greater observation-function complexity was associated with reduced identifiability and poorer multi-start parameter-estimation performance, although the consistency of this relationship varied among the predictors. By contrast, neither mean pairwise Jaccard similarity nor the standard deviation of the pairwise Jaccard similarities showed a consistent relationship with the investigated outcomes across templates.

Larger ratios of scale and offset parameters and smaller numbers of observables were associated with lower identifiability and poorer multi-start parameter-estimation outcomes. The ratio of summed observables showed a less consistent relationship, with coefficient directions varying more strongly across outcomes and templates. For dynamic-parameter identifiability, coefficients indicated lower identifiability with larger ratios of summed observables in 12 of 22 templates, with larger ratios of scale and offset parameters in 18 of 22 templates, and with smaller numbers of observables in 22 of 22 templates.

Figure S3 shows representative template-specific results for the two groups of outcomes: dynamic-parameter identifiability represents the identifiability outcomes, and best-observed-objective recovery represents the multi-start parameter-estimation outcomes. In addition to the coefficient directions summarized in Table S2, the figure shows the magnitude and uncertainty of the estimated relationships.

The magnitudes and uncertainties of the estimated relationships varied considerably among templates. Coefficients for the ratio of summed observables were often close to zero, with many confidence intervals including zero. Coefficients for the ratio of scale and offset parameters and the number of observables showed more consistent directions, although their magnitudes and confidence-interval widths also varied.

4.3 Discussion

The observation-function analysis indicates that greater observation-function complexity is associated with lower identifiability and increased parameter-estimation difficulty. The ratio of scale and offset parameters and the number of observables showed more consistent relationships with the investigated outcomes than the ratio of summed observables. In particular, lower observation-function complexity was generally associated with higher dynamic-parameter identifiability across template groups.

In contrast, neither mean pairwise Jaccard similarity nor the standard deviation of the pairwise Jaccard similarities showed a consistent relationship with the investigated outcomes. Observable co-occurrence, as summarized by these Jaccard measures, therefore did not systematically explain differences in identifiability or multi-start parameter-estimation outcomes.

Taken together, the investigated outcomes were more consistently associated with observation-function characteristics and the number of observables than with the investigated measures of observable co-occurrence. These results describe relationships within the variation represented by the existing benchmark collection.

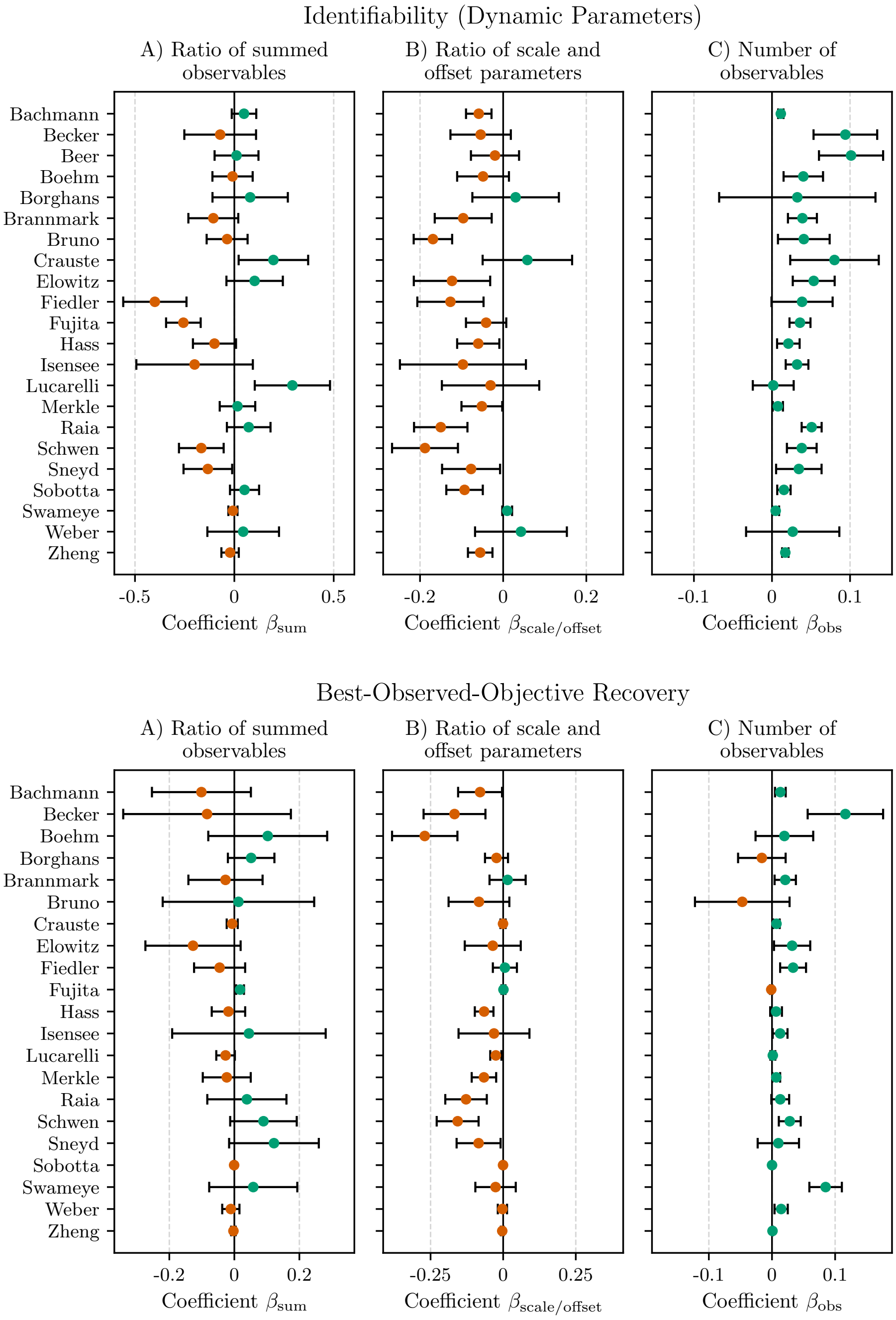

Figure S3: Template-specific regression coefficients for dynamic-parameter identifiability (top) and recovery of the best observed objective value during multi-start parameter estimation (bottom). These outcomes are representative examples of the identifiability and multi-start parameter-estimation outcomes summarized in Supplementary Table S2. Rows correspond to template groups; for recovery of the best observed objective value, the Beer template was excluded because the outcome was constant within this template. Panels A–C correspond to the ratio of summed observables, the ratio of scale and offset parameters, and the number of observables, respectively. Coefficients represent the change in the respective outcome fraction per unit increase in the corresponding ratio in panels A and B and per additional observable in panel C. Points show regression coefficients adjusted for the other two predictors. Negative coefficients are shown in red and positive coefficients in green. Horizontal error bars indicate 95% confidence intervals, and the vertical line marks zero. Higher ratios of summed observables and scale and offset parameters were associated with lower identifiability and less frequent recovery of the best observed objective value, whereas a larger number of observables was associated with higher identifiability and more frequent recovery.

References

J. Bachmann, A. Raue, M. Schilling, M. E. Böhm, C. Kreutz, D. Kaschek, H. Busch, N. Gretz, W. D. Lehmann, J. Timmer, and U. Klingmüller. Division of labor by dual feedback regulators controls JAK2/STAT5 signaling over broad ligand range. *Molecular Systems Biology*, 7(1):516, July 2011. ISSN 1744-4292. doi: 10.1038/msb.2011.50. URL https://www.embopress.org/doi/full/10.1038/msb.2011.50.

V. Becker, M. Schilling, J. Bachmann, U. Baumann, A. Raue, T. Maiwald, J. Timmer, and U. Klingmüller. Covering a Broad Dynamic Range: Information Processing at the Erythropoietin Receptor. *Science*, 328 (5984):1404–1408, June 2010. doi: 10.1126/science.1184913. URL https://www.science.org/doi/full/10.1126/science.1184913.

R. Beer, K. Herbst, N. Ignatiadis, I. Kats, L. Adlung, H. Meyer, D. Niopek, T. Christiansen, F. Georgi, N. Kurzawa, J. Meichsner, S. Rabe, A. Riedel, J. Sachs, J. Schessner, F. Schmidt, P. Walch, K. Niopek, T. Heinemann, R. Eils, and B. D. Ventura. Creating functional engineered variants of the single-module non-ribosomal peptide synthetase IndC by T domain exchange. *Molecular BioSystems*, 10(7):1709–1718, 2014. doi: 10.1039/C3MB70594C. URL https://pubs.rsc.org/en/content/articlelanding/2014/mb/c3mb70594c.

M. E. Boehm, L. Adlung, M. Schilling, S. Roth, U. Klingmüller, and W. D. Lehmann. Identification of Isoform-Specific Dynamics in Phosphorylation-Dependent STAT5 Dimerization by Quantitative Mass Spectrometry and Mathematical Modeling. *Journal of Proteome Research*, 13(12):5685–5694, Dec. 2014. ISSN 1535-3893. doi: 10.1021/pr5006923. URL https://doi.org/10.1021/pr5006923.

J. M. Borghans, G. Dupont, and A. Goldbeter. Complex intracellular calcium oscillations A theoretical exploration of possible mechanisms. *Biophysical Chemistry*, 66(1):25–41, May 1997. ISSN 0301-4622. doi: 10.1016/S0301-4622(97)00010-0. URL https://www.sciencedirect.com/science/article/pii/S0301462297000100.

M. Bruno, J. Koschmieder, F. Wuest, P. Schaub, M. Fehling-Kaschek, J. Timmer, P. Beyer, and S. Al-Babili. Enzymatic study on AtCCD4 and AtCCD7 and their potential to form acyclic regulatory metabolites. *Journal of Experimental Botany*, 67(21):5993–6005, Nov. 2016. ISSN 0022-0957. doi: 10.1093/jxb/erw356. URL https://doi.org/10.1093/jxb/erw356.

C. Brännmark, R. Palmér, S. T. Glad, G. Cedersund, and P. Strålfors. Mass and Information Feedbacks through Receptor Endocytosis Govern Insulin Signaling as Revealed Using a Parameter-free Modeling Framework*. *Journal of Biological Chemistry*, 285(26):20171–20179, June 2010. ISSN 0021-9258. doi: 10.1074/jbc.M110.106849. URL https://www.sciencedirect.com/science/article/pii/S0021925818496949.

F. Crauste, J. Mafille, L. Boucinha, S. Djebali, O. Gandrillon, J. Marvel, and C. Arpin. Identification of Nascent Memory CD8 T Cells and Modeling of Their Ontogeny. *Cell Systems*, 4(3):306–317.e4, 2017. ISSN 2405-4712, 2405-4720. doi: 10.1016/j.cels.2017.01.014. URL https://www.cell.com/cell-systems/abstract/S2405-4712(17)30044-3.

M. B. Elowitz and S. Leibler. A synthetic oscillatory network of transcriptional regulators. *Nature*, 403(6767): 335–338, Jan. 2000. ISSN 1476-4687. doi: 10.1038/35002125. URL https://www.nature.com/articles/35002125.

A. Fiedler, S. Raeth, F. J. Theis, A. Hausser, and J. Hasenauer. Tailored parameter optimization methods for ordinary differential equation models with steady-state constraints. *BMC Systems Biology*, 10(1):80, Aug. 2016. ISSN 1752-0509. doi: 10.1186/s12918-016-0319-7. URL https://doi.org/10.1186/s12918-016-0319-7.

K. A. Fujita, Y. Toyoshima, S. Uda, Y.-i. Ozaki, H. Kubota, and S. Kuroda. Decoupling of Receptor and Downstream Signals in the Akt Pathway by Its Low-Pass Filter Characteristics. *Science Signaling*, 3(132): ra56–ra56, July 2010. doi: 10.1126/scisignal.2000810. URL https://www.science.org/doi/full/10.1126/scisignal.2000810.

H. Hass, F. Kipkeew, A. Gauhar, E. Bouché, P. May, J. Timmer, and H. H. Bock. Mathematical model of early Reelin-induced Src family kinase-mediated signaling. *PLOS ONE*, 12(10):e0186927, Oct. 2017. ISSN 1932-6203. doi: 10.1371/journal.pone.0186927. URL https://journals.plos.org/plosone/article?id=10.1371/journal.pone.0186927.

J. Isensee, M. Kaufholz, M. J. Knape, J. Hasenauer, H. Hammerich, H. Gonczarowska-Jorge, R. P. Zahedi, F. Schwede, F. W. Herberg, and T. Hucho. PKA-RII subunit phosphorylation precedes activation by cAMP and regulates activity termination. *Journal of Cell Biology*, 217(6):2167–2184, Apr. 2018. ISSN 0021-9525. doi: 10.1083/jcb.201708053. URL https://doi.org/10.1083/jcb.201708053.

P. Lucarelli, M. Schilling, C. Kreutz, A. Vlasov, M. E. Boehm, N. Iwamoto, B. Steiert, S. Lattermann, M. Wäsch, M. Stepath, M. S. Matter, M. Heikenwälder, K. Hoffmann, D. Deharde, G. Damm, D. Seehofer, M. Muciek, N. Gretz, W. D. Lehmann, J. Timmer, and U. Klingmüller. Resolving the Combinatorial Complexity of Smad Protein Complex Formation and Its Link to Gene Expression. *Cell Systems*, 6(1):75–89.e11, Jan. 2018. ISSN 2405-4712, 2405-4720. doi: 10.1016/j.cels.2017.11.010. URL https://www.cell.com/cell-systems/abstract/S2405-4712(17)30538-0.

R. Merkle, B. Steiert, F. Salopiata, S. Depner, A. Raue, N. Iwamoto, M. Schelker, H. Hass, M. Wäsch, M. E. Böhm, O. Mücke, D. B. Lipka, C. Plass, W. D. Lehmann, C. Kreutz, J. Timmer, M. Schilling, and U. Klingmüller. Identification of Cell Type-Specific Differences in Erythropoietin Receptor Signaling in Primary Erythroid and Lung Cancer Cells. *PLOS Computational Biology*, 12(8):e1005049, Aug. 2016. ISSN 1553-7358. doi: 10.1371/journal.pcbi.1005049. URL https://journals.plos.org/ploscompbiol/article?id=10.1371/journal.pcbi.1005049.

V. Raia, M. Schilling, M. Böhm, B. Hahn, A. Kowarsch, A. Raue, C. Sticht, S. Bohl, M. Saile, P. Möller, N. Gretz, J. Timmer, F. Theis, W.-D. Lehmann, P. Lichter, and U. Klingmüller. Dynamic Mathematical Modeling of IL13-Induced Signaling in Hodgkin and Primary Mediastinal B-Cell Lymphoma Allows Prediction of Therapeutic Targets. *Cancer Research*, 71(3):693–704, Feb. 2011. ISSN 0008-5472. doi: 10.1158/0008-5472.CAN-10-2987. URL https://doi.org/10.1158/0008-5472.CAN-10-2987.

A. Raue, M. Schilling, J. Bachmann, A. Matteson, M. Schelke, D. Kaschek, S. Hug, C. Kreutz, B. D. Harms, F. J. Theis, U. Klingmüller, and J. Timmer. Lessons Learned from Quantitative Dynamical Modeling in Systems Biology. *PLOS ONE*, 8(9):e74335, Sept. 2013. ISSN 1932-6203. doi: 10.1371/journal.pone.0074335. URL https://journals.plos.org/plosone/article?id=10.1371/journal.pone.0074335.

A. Raue, B. Steiert, M. Schelker, C. Kreutz, T. Maiwald, H. Hass, J. Vanlier, C. Tönsing, L. Adlung, R. Engesser, W. Mader, T. Heinemann, J. Hasenauer, M. Schilling, T. Höfer, E. Klipp, F. Theis, U. Klingmüller, B. Schöberl, and J. Timmer. Data2Dynamics: a modeling environment tailored to parameter estimation in dynamical systems. *Bioinformatics*, 31(21):3558–3560, July 2015. doi: 10.1093/bioinformatics/btv405.

L. Schmiester, Y. Schälte, F. T. Bergmann, T. Camba, E. Dudkin, J. Egert, F. Fröhlich, L. Fuhrmann, A. L. Hauber, S. Kemmer, P. Lakrisenko, C. Loos, S. Merkt, W. Müller, D. Pathirana, E. Raimúndez, L. Refisch, M. Rosenblatt, P. L. Stapor, P. Städter, D. Wang, F.-G. Wieland, J. R. Banga, J. Timmer, A. F. Villaverde, S. Sahle, C. Kreutz, J. Hasenauer, and D. Weindl. PEtab—Interoperable specification of parameter estimation problems in systems biology. *PLOS Computational Biology*, 17(1):e1008646, Jan. 2021. ISSN 1553-7358. doi: 10.1371/journal.pcbi.1008646. URL https://journals.plos.org/ploscompbiol/article?id=10.1371/journal.pcbi.1008646.

L. O. Schwen, A. Schenk, C. Kreutz, J. Timmer, M. M. B. Rodríguez, L. Kuepfer, and T. Preusser. Representative Sinusoids for Hepatic Four-Scale Pharmacokinetics Simulations. *PLOS ONE*, 10(7):e0133653, July 2015. ISSN 1932-6203. doi: 10.1371/journal.pone.0133653. URL https://journals.plos.org/plosone/article?id=10.1371/journal.pone.0133653.

J. Sneyd and J.-F. Dufour. A dynamic model of the type-2 inositol trisphosphate receptor. *Proceedings of the National Academy of Sciences*, 99(4):2398–2403, Feb. 2002. doi: 10.1073/pnas.032281999. URL https://www.pnas.org/doi/abs/10.1073/pnas.032281999.

S. Sobotta, A. Raue, X. Huang, J. Vanlier, A. Jünger, S. Bohl, U. Albrecht, M. J. Hahnel, S. Wolf, N. S. Mueller, L. A. D'Alessandro, S. Mueller-Bohl, M. E. Boehm, P. Lucarelli, S. Bonefas, G. Damm, D. Seehofer, W. D. Lehmann, S. Rose-John, F. van der Hoeven, N. Gretz, F. J. Theis, C. Ehlting, J. G. Bode, J. Timmer, M. Schilling, and U. Klingmüller. Model Based Targeting of IL-6-Induced Inflammatory Responses in Cultured Primary Hepatocytes to Improve Application of the JAK Inhibitor Ruxolitinib. *Frontiers in Physiology*, 8, Oct. 2017. ISSN 1664-042X. doi: 10.3389/fphys.2017.00775. URL https://www.frontiersin.org/journals/physiology/articles/10.3389/fphys.2017.00775/full.

I. Swameye, T. G. Müller, J. Timmer, O. Sandra, and U. Klingmüller. Identification of nucleocytoplasmic cycling as a remote sensor in cellular signaling by databased modeling. *Proceedings of the National Academy of Sciences*, 100(3):1028–1033, Feb. 2003. doi: 10.1073/pnas.0237333100. URL https://www.pnas.org/doi/abs/10.1073/pnas.0237333100.

P. Weber, M. Hornjik, M. A. Olayioye, A. Hausser, and N. E. Radde. A computational model of PKD and CERT interactions at the trans-Golgi network of mammalian cells. *BMC Systems Biology*, 9(1):9, Feb. 2015. ISSN 1752-0509. doi: 10.1186/s12918-015-0147-1. URL https://doi.org/10.1186/s12918-015-0147-1.

Y. Zheng, S. M. M. Sweet, R. Popovic, E. Martinez-Garcia, J. D. Tipton, P. M. Thomas, J. D. Licht, and N. L. Kelleher. Total kinetic analysis reveals how combinatorial methylation patterns are established on lysines 27 and 36 of histone H3. *Proceedings of the National Academy of Sciences*, 109(34):13549–13554, Aug. 2012. doi: 10.1073/pnas.1205707109. URL https://www.pnas.org/doi/abs/10.1073/pnas.1205707109.
